# “They’re just shifting a problem”: A qualitative study of stakeholder perceptions of the welfare implications of UK rescue dog importation

**DOI:** 10.1371/journal.pone.0343416

**Published:** 2026-09-09

**Authors:** Lisa J. Wallis, Alisha Murphy, Gina Pinchbeck, Jenny Stavisky, Louise Buckley, Carri Westgarth

**Affiliations:** 1 Department of Livestock and One Health, Institute of Infection, Veterinary and Ecological Sciences, University of Liverpool, Liverpool, United Kingdom; 2 School of Veterinary Science, University of Liverpool, Liverpool, United Kingdom; 3 VetPartners, York, United Kingdom; 4 Deanery of Clinical Sciences, University of Edinburgh, Edinburgh, United Kingdom; Jaramogi Oginga Odinga University of Science and Technology, KENYA

## Abstract

The number of rescue dogs being imported into the UK has increased significantly in recent years but research into the welfare implications of this practice is lacking. The aim of this study was to investigate various stakeholder and owner experiences of behavioural and health problems in imported rescue dogs. Thirty interviews and four focus groups (totalling n = 54) were conducted with overseas and UK rescue representatives, trainers, transporters, veterinary surgeons, animal health officers, government representatives and dog fosterers and owners in the UK. In addition, eighteen online publicly available forum threads were selected using purposive sampling. Inductive coding of these data employing thematic analysis resulted in seven main themes. Related to health and behaviour the themes identified were: unfit for transport, welfare during transport, infectious disease, novelty, agency, and caregiver expectations. The final theme related to regulation and enforcement challenges with sub themes around: non-compliance, lack of resources, communication, jurisdiction and lack of cross-sector regulation. Overall, caregiver expectations often did not align with reality after the dog arrived, leading to costly surprises for new owners. Further, some dogs were felt to be not well-matched for the household and displayed challenging behaviours. The rescue organisation was perceived to have moral responsibilities to provide accurate information and pre-and post-adoption support, but this was felt to be inconsistently provided. Stakeholders perceive a number of health and behavioural welfare issues associated with importing rescue dogs, but preventing these is somewhat difficult due to the challenges in regulating and monitoring animal movements between countries. Greater education about expectations of imported rescue dog health and behaviour is recommended, alongside improved monitoring of rescue organisation and transport processes, in order to protect animal welfare.

## Introduction

Over recent years there has been an increase in the importation of dogs from abroad into the UK with the aim of rehoming them as pets. The proportion of owners who acquired their dog from abroad has increased from 4% in August 2020 to 8% in 2023 [1] reflecting an increase in Imported Rescue Dogs (IRD) acquired from a ‘UK rescue centre or rehoming centre for pets from abroad’. Limited research available suggests the increasing popularity of importing rescue dogs is driven largely by a combination of social media advertising, wanting to rescue rather than fuel breeding, and the belief that foreign dogs suffer more than dogs in UK rescue centres [2]. The dog and puppy trade is seen as not only a national, but an international problem, which would benefit from a united approach [3,4].

Only one published study has so far investigated the demographics and processes of overseas rescue dog importation to the UK [2]. The owner survey reported on 3080 dogs, which predominantly came from Romania (34%), Cyprus (22%), and Spain (19%). The majority were reportedly ‘street dogs’ (which could include stray and former pet dogs abandoned on the streets, dogs born on the streets and dogs taken from public and private shelters) (61%), others were rescued from cruelty (10%), relinquished by their owners (8%), were born in a shelter (4%), and a few were from the dog meat trade (1%). Owner understanding and reporting of correct importation processes was shown to be poor. Dogs which are going to be sold or rehomed (including imported rescue dogs, where a transfer of ownership takes place) must be transported into the UK via the Balai rules (Council Directive 92/65/EEC) and Trade in Animals and Related Products Regulations 2011, and the Welsh and Scottish equivalents, (collectively known as TARP) [5] for commercial importation and follow additional rules as compared to standard travel with an owner [6]. Commercial consignments which travel utilising non-commercial pet travel rules intended for pets travelling with their owners, are thus legally non-compliant.

The movement of animals such as pets poses known risks spreading infectious diseases including zoonoses [7]. A recent British Veterinary Association survey revealed 93% of veterinary surgeons were concerned about the import of rescue dogs from abroad and that 40% of companion animal vets have seen new or rare conditions associated with the importation of dogs [8]. Twenty percent (603/3080) of owners surveyed said their dog was imported to the UK with known health conditions and of these, Leishmaniasis was the fourth most common imported health condition (9.1%, n = 52) [2]. When respondents were asked if their dog had been tested since being in the UK, *Leishmania* positivity was reported in 14.8% (79/533). A 2009 study [9] found that most dogs in the UK with leishmaniasis (57%) were imported from Spain or had been resident there prior to UK entry. In 2018, during a welfare investigation of a UK rescue centre which predominately commercially imported and rehomed dogs from Eastern Europe, most implanted with Romanian microchips, *Leishmania* was identified in 10.5% (14/133) of dogs tested [10]. Further, of 113 tested for *Brucella canis*, two (1.7%) clinically healthy dogs were serologically positive [10]. Of 129 dogs tested for *Babesia canis*, three (2.3%, 3/129) of the dogs tested positive [10]. In the owner survey by Norman, Stavisky and Westgarth (2) Babesiosis was reported in 1.3 percent of those tested since being in the UK (n = 4/305), and Ehrlichiosis in 5.7% (n = 21/369). The first three confirmed clinical cases of canine hepatozoonosis in dogs imported into the UK were recently described [11].

There are also welfare concerns regarding behavioural issues with a study of UK owners reporting fearfulness/nervousness and escape behaviours in particular [2]. A Danish study [12] showed that former street dogs displayed fear, aggression, and stress more often than dogs reared from birth in Denmark. However, the difference between the two groups were small. Most veterinary surgeons (80%) felt that Danish owners were not well prepared to own former street dogs and most commonly reported them to struggle with fear of humans, stress, and problems when home alone.

Despite these observed welfare issues in some cases, rescuing dogs from overseas continues to be a popular action for many dog owners and animal rescue charities. However, in a 2023 poll conducted on the Veterinary Voices UK Facebook page (21k followers), 99% of veterinary professionals said they would not recommend rehoming a rescue dog from abroad [13]. There has been little published scientific investigation into the phenomenon of importing dogs from overseas into the UK in this is required in order to critically examine welfare and public health risks. New research evidence is required to enable evidence-based policy to be made in this increasingly high-profile area, in particular in relation to Brexit and future legislative changes.

The aim of this part of the study was to investigate the lived experiences of key stakeholders involved in importing rescue dogs and their perceptions of the impact on dog health and welfare (both for dogs rehomed from abroad and the domestic population) as well as understanding the roles of key stakeholders in this process. We specifically focused on IRDs, meaning those dogs specifically imported into the UK for the purposes of rehoming for ‘rescue’ purposes, rather than puppies or dogs bred commercially, although the difference can be unclear in some cases. The first objective was to conduct interviews and focus groups to assess the experiences of stakeholders, including police and border forces, local authorities, overseas rescues (English speaking only), veterinary professionals, owners, animal behaviour counsellors, transporters, and UK rescue charities impacted by caring for imported dogs. The second objective was to conduct an analysis of online forum posts and comments about behavioural experiences of adopting imported rescue dogs.

## Materials and methods

### Study design

A qualitative methodology was selected as most appropriate for the study aims. Qualitative methods such as in-depth interviews and focus groups are designed to investigate people’s experiences, behaviour and social relationships, including our interactions with animals, and the reasons why we choose to behave in certain ways [14,15]. They are particularly suited for understanding perceptions around health knowledge and behaviours [16]. They use relatively small samples to investigate individual experiences in-depth and with nuance, rather than aiming for a more generalised surface understanding [17].

### Positionality

The study was conducted by a multidisciplinary academic team, with a wide range of backgrounds, experiences and views regarding imported rescue dogs, including owning them and providing veterinary care to them. Online interviews were conducted by LW who is a female qualitative and quantitative postdoctoral researcher, non-vet with a background in animal behaviour, who has imported her owned dogs into the UK when moving job locations. Focus groups were conducted by LW and CW. CW is the Principal Investigator of the study, a non-vet female qualitative and quantitative researcher with a background in animal behaviour and human public health, who owns multiple rescue dogs including a rescue breeding dog thought to be from Ireland/Northern Ireland, and has also worked for a UK animal rescue charity. The selection and analysis of forum data was conducted by AM a veterinary undergraduate student interested in the topic, who also analysed the trainer/behaviourist focus group. The project was supported by collaborators GP, a female veterinary epidemiologist, JS a veterinary researcher who has worked in shelter medicine, and LB a veterinary nurse academic who owns multiple imported rescue dogs and runs a *Brucella canis* advisory service. The immediate data collection and analysis team (CW, LW and AM) entered the study with mixed views regarding importation of rescue dogs, all recognising both positive and negative aspects to the practice. The wider supporting team all had veterinary backgrounds and a diverse range of views and practices, generally either supporting or opposing the importation of rescue dogs. In summary, we expected to find a number of human health and animal welfare challenges regarding imported rescue dogs, in particular concerning behaviour and infectious disease, and a number of practices occurring around importation that impacted these outcomes both positively and negatively.

### Participant recruitment and data collection

Invitations to participate were distributed in multiple channels including letters published in Veterinary Record, Veterinary Times, distribution through corporate veterinary groups and independent vet groups and practices, British Small Animal Veterinary Association (BSAVA), the Association of Charity Vets (ACV), British Veterinary Association (BVA), Royal College of Veterinary Surgeons (RCVS), and social media platforms on Facebook and Twitter including the Veterinary Voices Facebook group. A recruitment survey was hosted on Qualtrics and obtained demographic and contact information for interested volunteers, and was open for a total of 54 days from 19/04/23 to 12/06/23. Participants were selected from this using purposive sampling in order to encompass a wide range of experiences and backgrounds. Participants were informed of the purpose and funder of the study.

**Fig 1:**
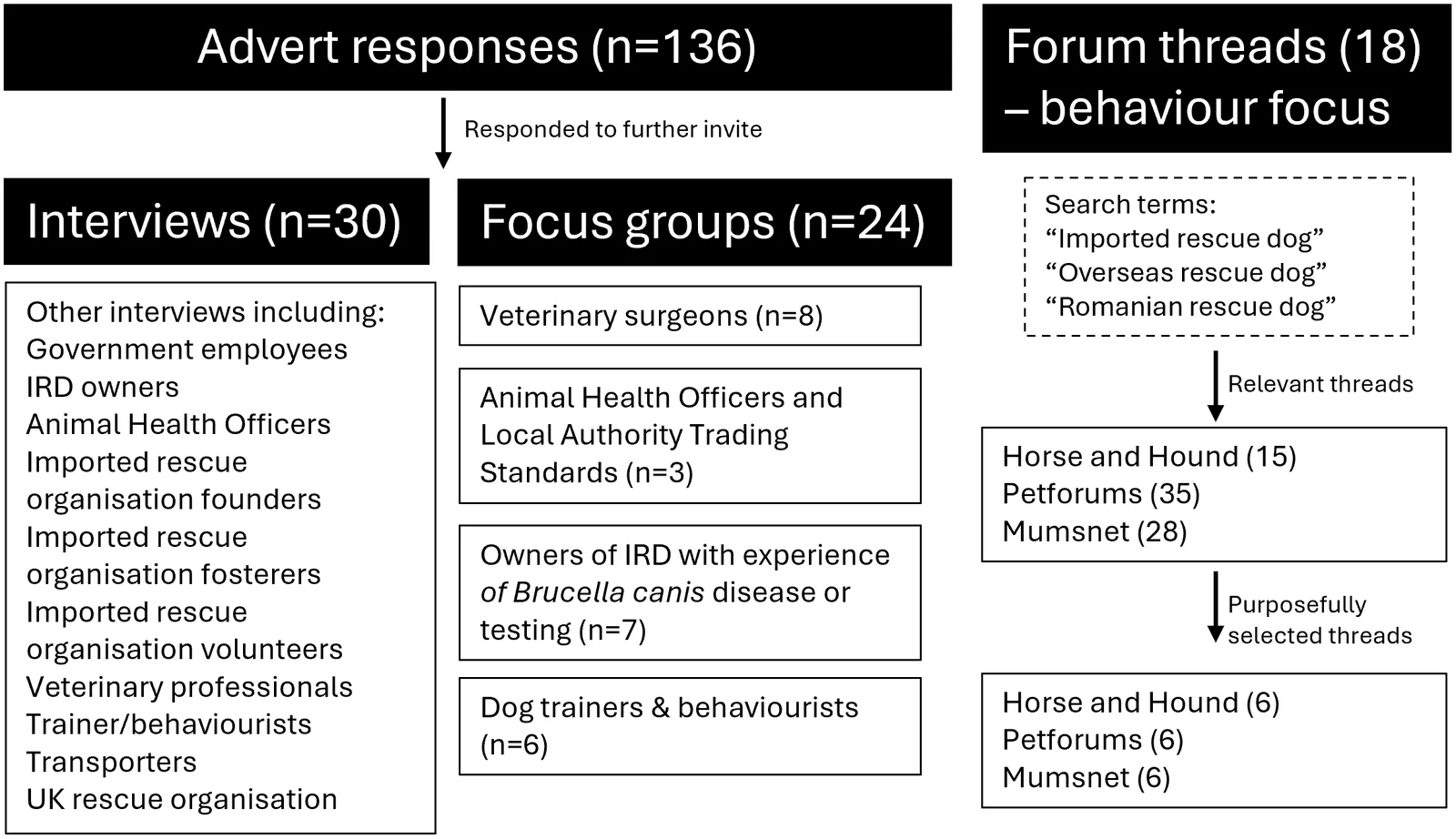
Overview of study data design and collection methods for 54 interview or focus group participants and 18 forum threads.

The study design and data collection is summarised in Fig 1. A total of 136 people responded to the online survey: after selection and follow-up for confirmed willingness, 54 contributed by online interview (n = 30) or focus group (n = 24). Four focus groups were conducted; one for veterinary surgeons (n = 8), one for dog trainers and behaviourists some of whom were expert witnesses (n = 6), one for local authority trading standards representatives and animal health officers (n = 3), and one for owners of IRD affected by *Brucella canis* policies or disease (n = 7). All participants are described in Table 1, and each individual could belong to more than one category. From initial recruitment data collected, the participants selected appeared to have positive views (15%), neutral/mixed views (11%), negative views (22%) or not provided (52%). Question schedules were developed by the study team in relation to the research needs and stakeholder group relevance (See S1 File). Field notes were used during/afterwards. Interviews varied in length from 30–134 (median 75) minutes and focus groups from 80–118 (median 109) minutes. In addition, eighteen threads (posts and their comments) from 3 publicly available online forums (Horse and Hound (6), Mumsnet (6), Petforums (6)) were identified through searches and selected using purposeful sampling, in order to encompass a range of IRD behaviours and contexts being discussed. Forum data were included as an additional accessible resource in order to encompass a larger sample of views around IRD and their behaviour than possible through interviews and focus groups alone. As the data is naturally occurring and not influenced by the presence of researchers it therefore may be less affected by social desirability bias which can affect surveys and interviews [18].

**Table 1:** Total number of participants in each stakeholder group category and country of import.

Category	Total	% of 54	Notes
Local authority Animal Health Officer/ Trading Standards	3	6	
Government employee	4	7	1 retired
Veterinary professional	12	22	11 veterinary surgeons (1 Government, 1 Quarantine), 1 veterinary nurse
UK rescue	4	7	All UK registered dog rescue charities
UK based overseas rescue founder	3	6	Location of import: Serbia, France, Romania, Spain
UK based overseas rescue volunteer	8	15	Location of import: Romania, Turkey, Bosnia, Spain, Serbia, Cyprus, Belarus
UK based overseas rescue dog fosterer	9	17	Location of import: Romania, Turkey, France, Bosnia, Spain, Serbia, Cyprus, Belarus
UK based overseas rescue dog owner	28	52	Location of import: Romania, Turkey, France, Macedonia, Bosnia, Spain, Serbia, Singapore, Croatia, Italy, Hungary
Dog trainer/behaviourist	10	19	3 work at UK based overseas rescues
Dog transporter	4	7	2 UK based overseas rescue (Spain, Romania), 2 private companies (Spain)

Please note each individual (n = 54) could belong to more than one category of stakeholder.

### Ethical considerations

The study was approved by the University of Liverpool Veterinary Ethics Committee (Project code VREC1233a). All interview/focus group participants provided written informed consent before the interview and verbal during. Internet forum participants did not consent but this is considered publicly available data appropriate for use by researchers.

### Data analysis

Audio recordings of the interviews/focus groups were transcribed either professionally or within Microsoft Teams. Thematic analysis of the interview/focus group transcripts (LW and AM) and discussion threads (AM) was conducted employing inductive coding managed in NVIVO software. Concurrent interviews and analysis were conducted until it was felt that a reasonable level of data saturation was reached to be able to present a number of clear themes and sub-themes [19,20]. Braun and Clarke’s (2006) six-phase process was used: 1) familiarising with the data; 2) generating initial codes; 3) searching for themes; 4) reviewing themes; 5) defining and naming themes; 6) producing the report [21]. Initial codes and themes were generated and similarities and differences identified by AM (forum analysis of behavioural concerns) supervised by LW and CW, who wrote her own report on these for her undergraduate studies. Separately and concurrently, LW analysed interviews/focus groups, supervised by CW, with further assistance from AM for analysing the behaviourist/trainer focus group. In order to challenge assumptions and consider alternative explanations, increasing rigor and further minimising the effect of the positionality of the researcher [22], the development of codes and themes were discussed in weekly conversations with CW; LW also attended the supervisory meetings of AM. Themes were then further refined in collaboration with the other authors during wider team discussions every 2–3 months and during drafting of the project report for the funder, which formed the basis of this paper. This enabled the creation of main themes, which encompassed the key subjects of the discussions, and sub-themes describing more detail within these. Quotes have been anonymised; given verbatim for those interviewed but slightly modified for ethical reasons for those taken from public forums in order to preserve anonymity. Due to time constraints, participants were not invited to provide feedback on transcripts or findings.

## Results

Eighty-three percent of interview participants were female and further demographic summaries other than Table 1 are not provided in order to preserve anonymity. Overall, participants were concerned that in some cases the priority of those involved in animal trade was not animal welfare.

*‘They’re not dog lovers, they’re movers of animals. They are just shifting a problem from point A to point B and making some money on it.’ (Local authority Trading Standards and Animal Health Officer focus group)*

**Fig 2:**
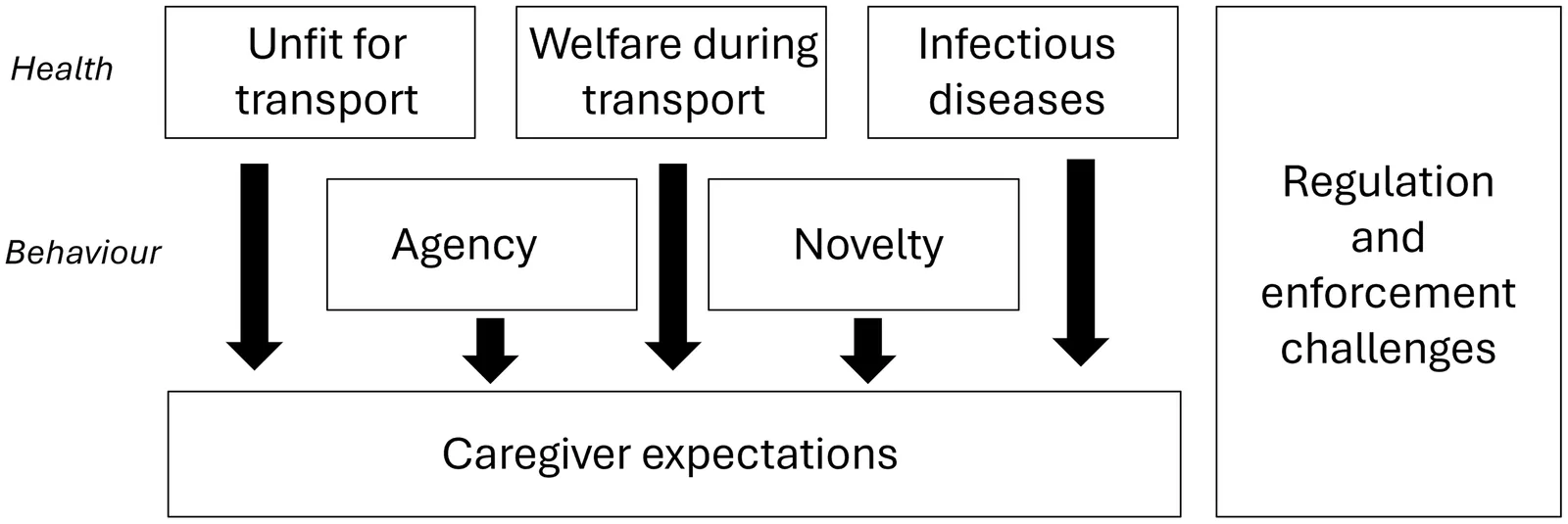
Main themes regarding stakeholder views of the welfare of imported rescue dogs.

Seven main themes (Fig 2) were created from the data. Related to health the themes identified were: unfit for transport, welfare during transport, infectious disease, novelty and agency. Related to both was caregiver expectations. The final main theme related to regulation and enforcement challenges.

### Health related themes

**Fig 3:**
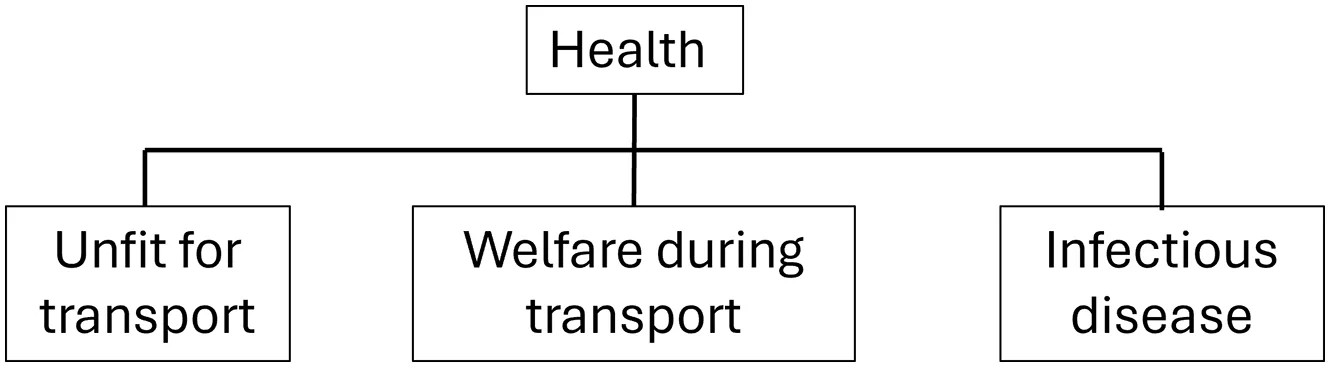
Health sub-themes regarding stakeholder views of the welfare implications of imported rescue dogs.

The overarching theme of health contained a number of sub-themes (Fig 3).

Whether a dog was **unfit for transport** was perceived as a grey area. Dogs were reportedly transported with recent surgical or open wounds, and pre-existing traumatic injury and disabilities, which were felt to contravene standards of being fit for transport by that participant, and/or according to transport guidance and legislation.

*‘Loads of them arrive with conditions which the new owners aren’t aware of. We had one that has been transported with a jaw fracture. It has been transported with multiple fractured teeth, and a deficit in its face, which wasn’t picked up by the vet. So, that raises concern about who’s doing the welfare checks on these dogs, and how are they deemed fit to travel?’ (Veterinary professional focus group participant)*

Clear illegal landings occurred when dogs were transported in the last 10% of their gestation period, as underage puppies, or if it was clear other non-compliance with health requirements had occurred.

*‘We’ve had bitches that have actually whelped down, as they’ve been transported, they’ve been that late in pregnancy.’ (Local authority Trading Standards and Animal Health Officer focus group)*

*‘He was passing live tapeworm when we got him, so we took him to the vet.’ (Overseas rescue dog owner interview participant)*

The theme **welfare during transport** included injuries, infectious disease and parasite spread, gastroenteritis, pancreatitis, dehydration, and poor body condition, either present before or caused/exacerbated by the process of transportation and reported by new owners on arrival.

*‘We had one who arrived to the new home with corneal ulcer straightaway. It’s likely to be travel related.’ (Veterinary professional focus group participant)*

*‘They were both in terrible condition, very underweight, dehydrated, diarrhoea, […] never smelt dogs this bad ever. Dull patchy fur, extremely nervous, ear infections and chronic digestive problems when arrived.’ (Overseas rescue dog owner interview participant)*

*‘So if anybody on [the van] has anything that is remotely contagious, you are then looking at a van full of dogs that could turn up to their new homes with issues.’ (Behaviourist/trainer focus group participant)*

There was also evidence of poor transport conditions uncovered at checkpoints such as unsuitable cages. Veterinary professionals reported that during transportation dogs often do not get fed for long periods, and water can also be withheld. Not all transport vehicles have air conditioning, and so the dogs may be subjected to extreme temperatures. None of the transportation companies interviewed reported using interventions such as sedatives or preparation through training.

*‘Quite often they’re transported in these so-called post buses that are vehicles that are bringing people and parcels from what’s mostly central and Eastern Europe to the UK, journeys easily of 20 or 30 hours. Generally, [they are] not fed because if you don’t put anything in you don’t get anything out. Very little water, no toilet stops.’ (Veterinary professional UK charity interview participant)*

*‘They’re not proper transportation crates, they are just your pet crates that they are being kept in. There’s a Dobermann pup in there, cropped and docked, coming through. This video will give you an idea of the size of the dog coming out of that crate. So, you can see that dog is not able to stand in its natural position.’ (Local Authority Trading Standards and Animal Health Officer focus group participant)*

The third health related theme was **infectious diseases**. Dogs were reportedly transported with exotic diseases including brucellosis and leishmaniasis, with sometimes owners and/or rescue staff aware and sometimes not. Some participants felt there was a lack of pre-import testing for exotic and zoonotic diseases needed to protect both animal welfare and public health. Veterinary surgeons emphasised the difficulty of persuading owners to carry out post import disease testing. UK rescues reported resource costs, welfare implications and human safety risks due to quarantine and barrier nursing required.

*‘We do the work for a charity that imports dogs from [country]. We have actually recently started testing for Brucella, and we’ve had two positive cases in the last few weeks, which is really scary, given that we’ve done lots of dentals and things on these dogs for months.’ (Veterinary professional focus group participant)*

*‘We’ve got a local charity who knowingly imports Leishmania dogs. They say that they’re cheap to treat, and that’s their argument for bringing them over.’ (Veterinary professional focus group participant)*

*‘At one point there was a puppy that was being barrier nursed until we had the [Brucella] test results. There is a welfare impact to these animals if they’re already in our care and then we find out they’ve been overseas, then you know this puppy was having people in PPE. There’s a welfare impact and a resource impact,’ (UK rescue organisation interview participant)*

### Behaviour related themes

**Fig 4:**
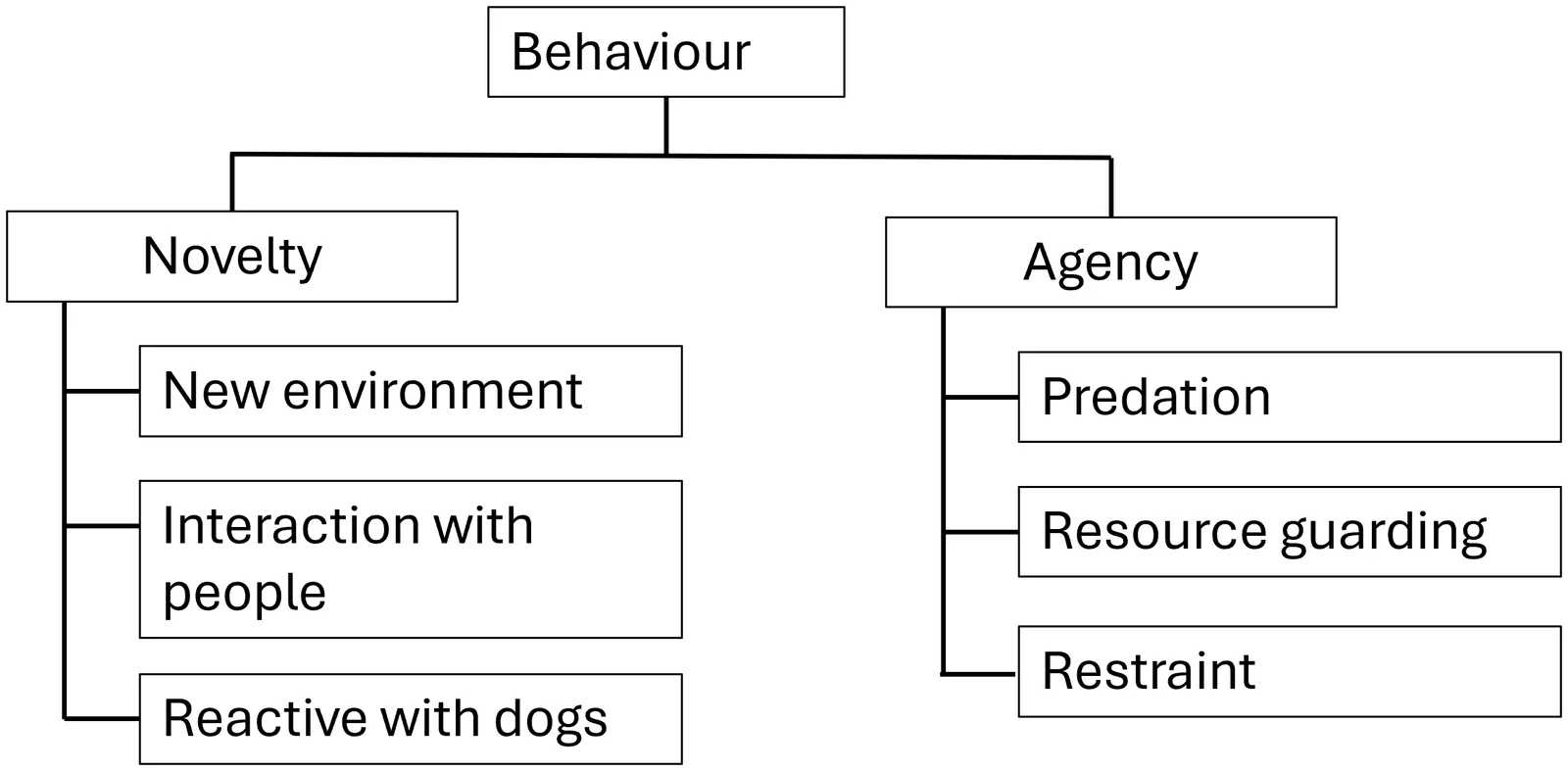
Behaviour sub-themes regarding stakeholder views of the welfare implications of imported rescue dogs.

There were a number of sub-themes under behaviour (Fig 4).

The first theme related to behaviour was **novelty**, as some dogs were reported to encounter difficulties with new environments, objects or strangers. Owners reported dogs often displaying nervous behaviour regarding their **new environment**, often thought to be the first time the dog has ever lived in a home.

*‘He was terrified of household noises, the TV, traffic’ (Forum participant)*

*‘It’s so sad, she is only a baby, and she spends all her time hiding in the crate, as she so scared, she won’t come out’ (Forum participant)*

*‘Many overseas rescue dogs are former street dogs and have never lived in a house, been in a car, or walked on a lead.’ (Forum participant)*

The second sub theme of novelty was **interaction with people**. Generally, dogs were felt to eventually get used to their owner’s presence, although this could take a long time, but they could still struggle with strangers or males. Wariness of people made seeking veterinary care difficult in some cases, including resulting in a bite risk to veterinary professionals.

*‘Once he got to know people, he was loving but he was really wary of strange men in particular’ (Forum participant)*

*‘First time I took him to the vet he bit her’ (Forum participant)*

*‘My foreign caseload is mainly dog-to-person aggression.’ (Behaviourist/trainer focus group participant)*

**Reactivity to other dogs** was also problematic in some cases, and was shown typically towards unknown dogs, but also cases of aggression towards other dogs in the household were found.

*‘When we first got him, we couldn’t go near the park, as he got very upset if he saw another dog.’ (Forum participant)*

*‘When we first got him, he would play with my other dog on walks, but then a few weeks later, he attacked her. After that he would growl at her if she tried to come in the living room. She was very submissive towards him, but he wouldn’t stop, and one day he attacked again, luckily while we had him on the lead. But I could see she was terrified of him. It was then I knew we had to take him back to the rescue.’ (Forum participant)*

The second behaviour theme was **agency**, describing how some dogs found the lack of control over their new environment and their inability to choose for themselves challenging, resulting in escape, difficulties being confined, **resource guarding** and **predation**. Some of these dogs were thought to be discarded hunting dogs and this was used to explain the high prey drive of certain individuals.

*‘Having been a stray they can be aggressive over resources such as food and toys’ (Forum participant)*

*‘The dog killed their cat which is just awful.’ (Forum participant)*

*‘’As many have been discarded by hunters, they will chase anything they can.’ (Forum participant)*

Challenges dealing with **restraint** was also a sub-theme of agency. Some owners struggled with using physical restraint tools, such as leads and collars. Owners reported some dogs to feel more secure by confinement such as a crate, whilst others struggled with it. Dogs were described as having a desire to escape from restraint and confinement, including jumping out of gardens over fences, or from transport vehicles.

*‘He freaked out when I put the collar and lead on, bolted, then attempted to jump our 5ft garden fence.’ (Forum participant)*

*‘We have seen it all on social media, where the dogs come over, it has been handed over at a service station, and the next day it is run over because it made an escape from transport.’ (Behaviourist/trainer focus group participant)*

*‘I am worried about leaving him alone as when we left him overnight downstairs we came down to absolute destruction. I tried to leave him in a crate, but he got so upset.’ (Forum participant)*

### Caregiver expectations

**Fig 5:**
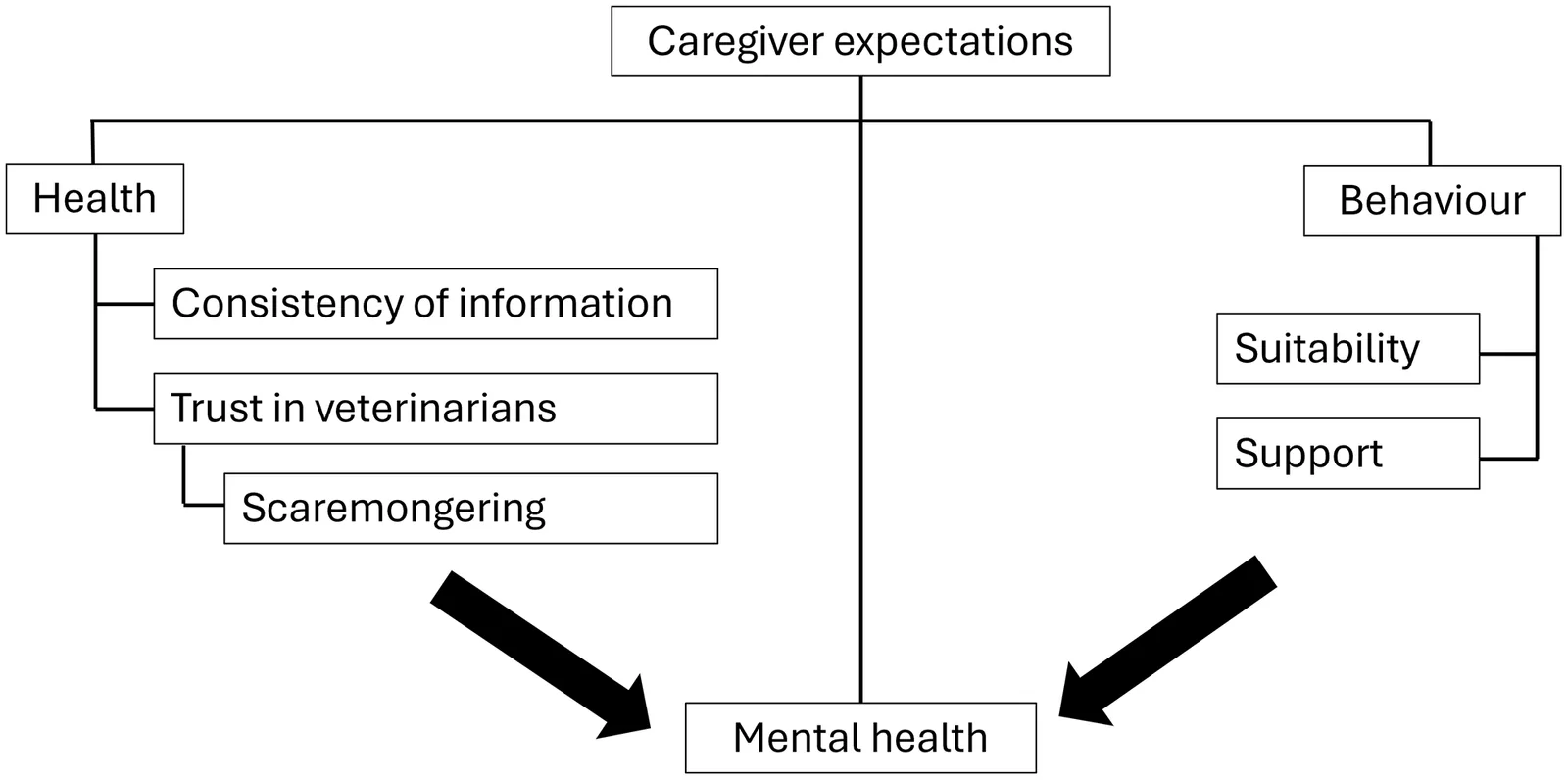
Caregiver expectations and sub-themes regarding stakeholder views of the welfare implications of imported rescue dogs.

A major theme across health and behavioural aspects was **caregiver expectations** (Fig 5), as these often did not align with reality after the dog arrived and led to costly surprises. This included infectious disease screening and extensive veterinary care or quarantine costs, or extensive behavioural support.

The availability and **consistency of information,** in particular about exotic and zoonotic diseases, varied widely. Owners reported that they were not always given correct information from the rescue organisations. It was perceived that some pre-import tests are not reliable, and/or rescues overseas may falsify test results, as most are not reported officially, and often only consist of a photograph.

*‘So I spoke with our vet and the vet said well if you want to report it, if it is found to be falsified documents and then he is going to be quarantined. […] So it’s four months quarantine at a cost of about £3000. […] Since then, I’ve just been trying to raise awareness of it and along the way I realised as well that there’s no support for people like me who fall victim to this.’ (Overseas rescue dog owner interview participant)*

*‘[It] came back positive. […] [The rescue] swore blind that he was negative. […] This is our second round of the […] treatment for him. […] If I’d known, cause it’s so expensive, you don’t envisage a disease from another country, I suppose. We expect normal vet bills. I didn’t expect this cause every time it’s about £1000.’ (Overseas rescue dog owner interview participant)*

Some veterinary professionals have recently insisted on post import disease testing of rescue dogs, even though they are already living in the UK and have been receiving veterinary care at the clinic for up to 10 years and show no signs of disease. Some owners were refused veterinary treatment for their dog if they had not been tested (for *Brucella canis*). Many were not advised of outcome options before the tests occurred and then some were advised to euthanise.

*‘They’re telling us to put our healthy dogs down. I mean my dog’s got no sign of Brucella. She’s perfectly healthy. I do think the vets need a lot more knowledge before they’re telling us to just euthanise your dog.’ (Overseas rescue dog owner affected by Brucella canis focus group participant)*

*‘They are basically saying they will provide emergency care only until the testing happens or after the test. They haven’t yet said what they will advise if the test comes back positive.’ (Overseas rescue dog owner affected by Brucella canis focus group participant)*

These types of experiences were attributed to the sub-theme **trust in veterinarians**. Mistrust from UK professionals was aimed at overseas veterinary surgeons and rescues who are thought to falsify information or provide inadequate vaccine dosage for financial gain. Because of this mistrust, owners reported increasingly being asked to pay for vaccinations and tests that they did not think they needed resulting in reduced trust in UK veterinary surgeons and companies, as participants perceived them to be trying to make a profit.

*‘We found the vets were not accepting our serology tests and were insisting that it was done again, and they wouldn’t see them in the practice or treat them in the practice until the tests were redone.’ (Overseas rescue dog owner and rescue volunteer affected by Brucella canis focus group participant)*

*‘It’s large practices that’s asking for it [testing] that seem to be more based on profit because they’re owned by some big faceless organisation. […] You know, once upon a time, you would trust your vet implicitly on all things, but bit by bit it’s been eroded.’ (Overseas rescue dog owner affected by Brucella canis focus group participant)*

After the first dog to human transmission of *Brucella canis* in 2022, owners, rescues and veterinary professionals became increasingly concerned about the perceived zoonotic risk of Brucella, and some participants perceived **scaremongering** occurred, with sources of evidence-based information difficult to find. Stakeholders felt that a clearer explanation of the risk of false positives and negatives according to testing protocol and the likelihood of infection according to the dog’s origin and life history was needed.

*‘When the story of the lady hit the headlines that she had been hospitalised with Brucella. The adopters started contacting us, saying “what’s going on here?”. We’re really panicked. So I had to start gathering as much information as I can. I tried to go to as many reliable sources rather than the scaremongering that seemed to be going on.’ (Owner and rescue volunteer participant)*

‘*Results need to be given in a quantitative way as well so that we can see whether there is a likelihood of false positives. So we can request additional testing.’ (Focus group for owners of overseas rescue dogs affected by Brucella canis)*

Owner expectations of the dog’s behaviour also often did not align with reality after the dog arrived. This was often felt to be due to the dog’s **suitability**, not being well-matched for the household or lifestyle or displaying unexpected behaviours. Some perceived being misled by the rescue centre, or not being adequately prepared or supported by them. The experience of the owner specifically with imported rescue dogs was also perceived as an issue. A combination of all these factors meant that owners often had unrealistic expectations and when these were not met it could eventually lead to a desire to relinquish the dog.

*‘The OH didn’t know they were from Romania until after he’d paid for them, and we received a magazine saying how they rescue the dogs from there.’ (Forum participant)*

*‘The rescue must take responsibility for their dishonesty and can’t blame you. They gave you a dog with challenges even though they knew you were inexperienced and had kids in the house.’ (Forum participant)*

*‘I think the dog should be coming over into foster homes here with experienced people that can fully assess those dogs. […] You could have a great dog over in Romania, you drop it off to a family that has got four crazy children, and the dog immediately is not in a good place.’ (Behaviourist/trainer focus group participant)*

Some UK dog rescues stated that the importation of rescue dogs which were not suitable for rehoming into UK families has resulted in an influx of animals into UK rescues which are already full and impacted dog welfare.

*‘’There are so many dogs over here now hundreds every day in the UK kennels that are getting put to sleep. Because there’s nowhere for them to go. Why on Earth are they bringing any more over? It doesn’t make sense.’ (Owner and IRD fosterer interview participant)*

The rescue organisation was perceived to have moral responsibilities to provide accurate information, and **pre-and post-adoption support** was deemed crucial. Often this was provided through online support groups, but other support was sometimes lacking, and then fell to UK based rescues and other animal professionals to provide.

*‘The organisation I use will always take the dog back and have a great support group on Facebook’ (Forum participant)*

*‘In the paperwork they explained what to expect and they are likely to be frightened of noises and household objects.’ (Forum participant)*

*‘If their rescue doesn’t have a behaviourist attached to them, they have then got to look at paying for a behaviourist to come and help them. I think it is an awful lot for people to take on.’ (Behaviourist/trainer focus group participant)*

*‘Rescue backups. So, say that they come in and it doesn’t work out. There’s often then nowhere for them to go because [the rescue is] online based or they’ll be foster network based. So, then that’s when we get contacted and they’re like we can’t get hold of the charity or they don’t have anywhere for them to go, so they’ve told us to contact elsewhere.’ (UK rescue organisation interview participant)*

The final sub-theme under caregiver expectations was **mental health impact**. In particular, owners described how their mental health was affected by waiting for health test results, where a positive result could mean euthanasia. This also impacted UK rescues and other stakeholders who for example had to euthanise whole litters born into their care. Some owners also experienced the dog’s behavioural problems to be too difficult and could not cope, leading to mental health impacts, and potentially relinquishment, euthanasia, or conflict with the rescue organisation.

*‘I was thinking if one of them tests positive I could lose all my dogs in one go. The mental stress was almost intolerable. […] The mental impact on all of us, is horrendous and that could have been changed, I think in the way it was handled.’ (Overseas rescue dog owner affected by Brucella canis focus group participant)*

*‘“I mean there have been times I’ve just wanted to give up and I’m just thinking I can’t do this. I really can’t do this because, you know, I wasn’t told about these issues.’ (Overseas rescue dog owner interview)*

*‘I am aware of ones where caregivers have been in agreement with the welfare decision to euthanise and have been unable to implement that decision because they’ve been interfered with by the organisation, and the dog has been removed from their care. But where those dogs have gone after they’ve been removed from their care, I don’t know. And I don’t think the caregivers know.’ (Dog behaviourist interview participant)*

Nevertheless, where owners and caregivers were willing and able to commit to the IRD, they found the experience rewarding, despite the difficulties encountered. Hard work, patience and long-term commitment were recognised as necessary by owners to progress and reach a suitable standard with their IRD, and for some the journey was ongoing.

*‘When he first arrived, he was worried about everything, but with time and patience he has expanded his comfort zone, and it has been wonderful to watch him grow.’ (Forum participant)*

*“Most rewarding thing I have ever done. I love my two to bits. I have had them for 6 years now and every time I look at their happy faces I am filled with gratitude. It’s been far from easy, but I wouldn’t change them for anything.” (Forum participant)*

*“If you give them the right home, where they have got time, patience, love and kindness. They will eventually turn around. […] As long as they have a relationship with someone, a bond with someone, they turn out to be really nice pets for those people and those homes.” (Dog behaviourist interview participant, charity volunteer and owner).*

### Regulation and enforcement challenges

Participants felt that despite there being some regulations applying to movements of dogs, these were ineffective, very challenging to police, and to monitor and trace movements that are happening or upcoming.

*‘In my opinion the importation of rescue dogs is problematic, and it is shocking how many rescues in the UK or in the country of origin are not fully familiar with legislation or are willing to bypass legislation to find a loophole.’ (Forum participant)*

*‘Certainly, with cows, it’s a legal requirement for every time it moves to inform the database. If we had the same thing with dogs, we might have a bit of a chance.’ (Local authority Trading Standards and Animal Health Officer focus group)*

**Fig 6:**
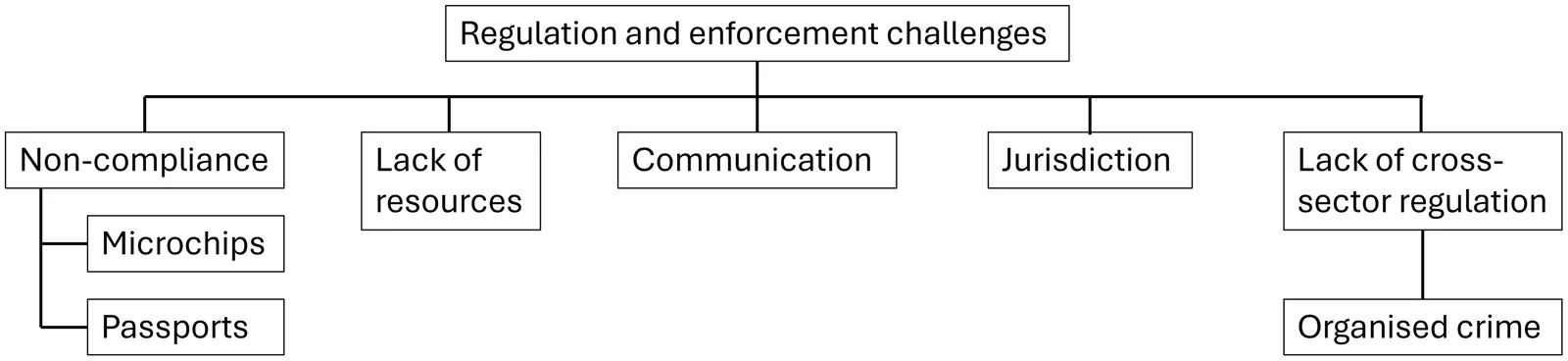
Regulation and enforcement challenges and sub-themes regarding stakeholder views of the welfare implications of imported rescue dogs.

Cases of non-compliance were raised, and regulation and enforcement were seen to be challenging due to a lack of resources, jurisdiction, cross sector regulation and problems with adequate communication between stakeholders (Fig 6).

A major theme with many stakeholders was **non-compliance**. Dogs were transported without the correct paperwork, with incorrect documentation, or with falsified documents.

*‘We believed he was from Romania, but we subsequently found out that he wasn’t. He was from Serbia on falsified documents.’ (Overseas rescue dog owner interview participant)*

*‘We’ve seen a massive, massive increase in fake passports, fake health certificates. I’ve never seen anything like it, but it’s just exploded.’ (Government employee interview participant)*

*‘I would think at least 30% of everything that comes into this country is in one form or another, not fully legal.’ (Government employee interview participant)*

A particular challenge to monitoring dog movements is how **microchips** and **passports** are open to misuse.

*‘You can put the microchip- stick it with a bit of Sellotape onto a collar, and as soon as you’ve got into the UK take it off. Or you don’t even have to put it on the collar, as long as it’s in the bottom of the pen somewhere, where it can be scanned as they run the scanner around.’ (Local authority Trading Standards and Animal Health Officer focus group participant)*

*‘If we had one centralised system where all microchips were registered, then that would be a massive help to the whole of this industry.’ (Local Authority Trading Standards and Animal Health Officer focus group participant)*

*‘They stopped consignment boxes full of passports already fully completed with the microchips, ready to go into these dogs […] It’s all fully completed, all signed by the vet.’ (Local Authority Trading Standards and Animal Health Officer focus group participant)*

One huge challenge to enforcement was the theme of **lack of resources** (including staff, premises, and time). In order to be effective, there was a perceived need for higher controls at border posts, but these were not currently resourced, leaving clear gaps.

*‘We were a 24/7 agency, we were doing days and nights and then [due to] government cutbacks […] then just worked Monday to Friday […] It made it more difficult and probably made us slightly less effective than we should have been.’ (Government employee interview participant)*

*‘There will be a small animal border control post at some point and then hopefully lots of the commercial trade will have to go that way to be checked. But we haven’t got there yet. [After Brexit] We haven’t got any operational border control posts for roll on for the ferry trade yet.’ (Government employee interview participant).*

Other pressures such as COVID and the Ukraine war further contributed to challenges with checks at the border.

*“Because the demand was there but people actually couldn’t go and view puppies and buy them, but they could click and buy something online that would be delivered to their door, like an Amazon parcel. And so that seemed to be what happened during lockdown. It was just ridiculously busy, an explosion of imported dogs and cats.” (Government employee interview participant)*

*“But then something like Ukraine happens. And then my job then has to focus on helping the Ukrainians with their pets, making sure they get into quarantine. We were not able to spend so much time looking for illegal imports because we were having to deal with the real people crisis.” (Government employee interview participant)*

One loophole that was revealed which allows dogs to be imported into the UK via a commercial transporter, but remain under the non-commercial pet travel rules documentation intended for non-commercial animals, was the use of **owner declarations**, which was felt to be increasing and easily faked.

*‘That has been a huge game changer because now people realise just how easy it is to bring the dogs in. […] I hear some of them were even buying cheap flights tickets for £17.00 to prove that the owner was travelling within 5 days of the dog. And so, it doesn’t really matter what measures you put in place they will find a way.’ (Owner interview)*

*‘The animals are being taken and declared as being non-commercial pets. It’s very easy thing to do. You only need the pet passport and the owner’s declaration, which is a one page, DEFRA form which can be simply forged with any name you care to make. […] because there is zero follow up and there never has been any follow up, simply by showing that at the desk at Eurotunnel you’re in and then you disappear. Dog disappears.’ (Transporter interview)*

There were also issues identified with the systems for recording commercial movements, in particular due to lack of replacement EU border control posts post-**Brexit**. The correct original origin country outside the EU may not be included on the documentation for dogs entering the UK from areas of high rabies incidence into the EU first.

*‘If you’ve got rescue dogs or puppies born in Serbia, Serbia is unlisted, so you would need to have the animals vaccinated at 12 weeks, wait 30 days, get a blood test, and then wait three months. So you’ve got the youngest an animal can come in from Serbia is 7 months now. A 7 month old puppy doesn’t hold as much value as a, you know, 15 week old puppy. So what they do is and we’ve just had Intel this week actually about this bus load every week. Busload of dogs come from Serbia via Croatia into Hungary. In Hungary they’re given Hungarian documents, so they’re microchipped given Hungarian EU passports, vaccinated, […] made to look like they are of Hungarian origin.’ (Government employee interview participant)*

In addition, the implementation of **post import checks** were felt to be insufficient. When non-compliance is detected during these checks, animals may be seized and put into quarantine due to disease risk. Due to the costs of quarantine some dogs are abandoned. This was felt to impact not just animal welfare, but the wellbeing of the owners and workers involved.

*‘So, these three dogs had been with the owners for a good four to six weeks, before it was picked up on. So, those dogs had started bonding with their new owners, and then I had to go and take them off them and put them in quarantine for three months. So, we have to start the process again. So, in terms of the welfare of the dogs, it’s awful.’ (Focus group Animal Health Officer participant)*

*‘There’s no government pot. Quarantine kennels. They’re all private and private companies. So, then [they] were left with these dogs in quarantine. […] They shouldn’t be putting their hand in their own pockets for this, but they do it because they genuinely care for the animals.’ (Government employee interview participant)*

Targeted interventions to protect health and biosecurity have been used, including the **safeguarding ban** where commercial import of dogs, cats and ferrets from Belarus, Poland, Romania or Ukraine (a country which can pose a rabies risk) was temporarily suspended in April 2022, and after the ban was lifted in October 2022, the launch of the new ‘**Approved Importers Scheme’** [23]. However, participants felt that the same problems would persist.

*‘As the ban went on, you saw them hone their methods, you know, and they were getting more efficient at it because when it started, you know, it was all a bit slapdash and people going to Ireland to try and come in or, you know, people dropping dogs at car parks in Calais. By the end of the ban, they had rescues in France who were acting as the destinations for people to then go and collect the dogs from.’ (Owner interview participant)*

*‘I don’t think [the Approved Imported Scheme] is going to solve the problem because those that are genuine will try their hardest and will register. But those that have found a viable way to get round the regulations will continue to do so. They won’t register. They’ll just carry on as they were.’ (Government employee interview participant)*

Another theme was issues with effective **communication**, which included communicating intelligence between agencies for targeting incoming consignments for checks, and also communication of information between agencies, rescue organisations and owners.

*‘[TRACES has] been replaced by our own system called IPAFFS. In an ideal world, the agent puts on the notification and uploads all the documents and gives you a heads up of when the dog or cat or whatever it might be is coming in. But it’s a fairly new system and it’s not very robust. If people don’t upload their health certificates, etcetera ahead of the arrival, it’s not always noted.’ (Government interview participant)*

*“Quite a few local authorities at that time had got read-only access to TRACES. Then, that would at least allow you to see what was coming into your area. You could search by post-code, and all that has now gone.” “We’ve got none of that with IPAFFS. That intelligence footprint isn’t there.” (Local authority Trading Standards and Animal Health Officer focus group participant)*

*‘And [the rescue] was telling us it was just a paperwork mix up. The trading standards were telling us it was because of the risk of rabies. And we were just not getting the information. Not just me, but all the other people they were getting mixed information from APHA, from Defra, from [the rescue]. None of us knew who to believe.’ (Owner interview participant)*

Whilst the systems and methods for illegal and fraudulent activity are widely known, routes and sanctions to tackle perpetrators based outside of the UK are limited due to reliance as these individuals may fall outside of UK **jurisdiction**.

*‘By the time we’re involved, put an animal into quarantine, the animal has been landed quite often. It’s been landed by a third party who’s probably a non-UK resident, so again, very difficult to get that person so that we can look at taking action against them. […] It has to be the person who is physically landed it not Mrs Miggins, who I found in the middle of Staffordshire who’s got this animal by the time we get involved.’ (Local Authority Trading Standards and Animal Health Officer focus group participant)*

A major theme identified across all stakeholders interviewed, was the issue of **lack of cross-sector regulation**. It was felt that is impossible to be able to effectively regulate one small part of the animal rescue community, those organisations importing from overseas, given that locally, animal rescue centres and other animal professions are not regulated or licensed either.

*‘There’s no regulation, don’t have to do anything. You don’t have to tell HMRC. You don’t have to declare anything. I can literally go on Facebook and say I’ve got this dog in a public shelter in Romania, send me donations, and people will send you money. Here’s my PayPal and off you go.’ (Overseas rescue founder interview participant)*

*There must be regulation. That is the only way forward, and I think, there is so much to regulate. […] Much of the business we all work in, there is no regulation. Anybody tomorrow could say they are a behaviourist or a trainer and set up a business and go for it.’ (Behaviourist/trainer focus group participant)*

The lack of regulation and cross-sector communication was reported to allow individuals to raise large sums of money through social media, which may not be declared, and even links to **organised crime** both in the UK and overseas were reported by stakeholders.

*‘The importation of a rescue is the equivalent of in effect buying a dog from abroad, buying goods and as imported goods import VAT does apply at 20%, and it’s those monies which are not finding their way to HMRC as best I can see. For example, you could declare a dog on IPAFFS quite correctly, but HMRC wouldn’t even know a dog was arriving and therefore that monies are due. So there’s very little joined up government on this one.’ (Transporter interview participant)*

*‘The problem is it’s organised crime, you’ve got, drug dealers turned to dogs because you know it’s more lucrative with far less risk. And we’ve got proof of this. […] No one seems to take it seriously, and that’s what we’re working on at the moment to try and build police links and links with border Force and HMRC.’ (Government employee interview participant)*

## Discussion

In line with previous research, this study identifies a number of health and behavioural welfare concerns regarding importation of rescue dogs into the UK. A key finding of this in-depth investigation of stakeholder views was that owner expectations about their imported rescue dog’s health and behaviour sometimes did not align with reality after their dog arrived. It was felt that rescue organisations needed to provide more accurate information, better matching processes and greater adopter support. Many stakeholders were fearful of zoonotic risk, and communication and testing challenges around this also led to a significant impact on caregivers’ mental health. Participants felt that current commercial and non-commercial transport regulations for companion animals were ineffective and difficult to enforce and to monitor, with illegal transportation/imports common which has implications for animal welfare. The biggest perceived challenge to effective enforcement was lack of resources, in particular a lack of adequate live animal border inspection facilities at ports and the presence of loopholes enabling circumnavigation of regulations. Increased regulation of imported rescue dogs was considered to be in dire need, but challenging considering there is no wider underpinning regulation of UK rescue organisations or wider animal sectors.

### Health

Many rescue organisations transport dogs with known and unknown health issues, for a complex mixture of reasons, likely including market forces and potential availability of suitable homes and veterinary care in the UK. Concerns about fitness to travel of these animals were raised including that they were likely to have experienced pain and discomfort at some point during transportation in, in contravention of legislation designed to prevent unnecessary suffering such as (EC) No1/2005 and The Welfare of Animals (Transport) (England) Order 2006 and parallel national legislation in Scotland, Wales and Northern Ireland.

International movement of animals raises concerns as the journey is likely to be long, with few rest periods. Challenges include stress and anxiety, physical discomfort, lack of access to food and water, fluctuations in temperature and ventilation, and disease transmission [24–26]. We found little evidence that interventions that would alleviate some of the stress experienced by dogs during transportation [27] typically took place, and evidence of unsuitable transport environments.

Since IRDs can be imported with exotic and zoonotic diseases which can be passed onto dogs and owners in the UK, this adds an extra dimension of welfare impact. Many stakeholders were concerned about these diseases and highlighted the lack of pre or post-import testing and that owners could be reluctant to test their dogs. Infectious diseases pose safety risks to human handlers of IRDs and handling protocols and testing also incur costs for UK rescue organisations when IRDs later come into their care and dogs which test positive may be euthanised. The negative impacts of euthanasia on veterinarians and dog caregivers are well known and can include psychological distress and burnout [28–31].

### Behavioural issues

The ability of IRDs to adjust to their new home can lead to behavioural challenges. Dogs were found to struggle with novelty and lack of control in their new environment, probably being used to greater agency. Our findings align with previous studies which also reported nervousness, aggression and escape behaviours [2,12]. In addition, many dogs which are imported into the UK, even under the guise of rescue, are actually sourced from high volume puppy farms in central and Eastern Europe and Ireland [32]. Previous studies have reported the long-term behavioural issues of these dogs, which are often sold via the internet or through brokers [33–36].

### Owner expectations

When purchasing a dog, consumers are often lacking in knowledge about the responsibilities and costs of dog-keeping with less than 20% of purchasers reporting being well informed about animal welfare and health at the time of purchase [37]. This issue was also evidenced regarding owner expectations of health and behaviour when adopting IRDs. This is not specific to imported rescue dogs though as owner expectations has been found as an issue in wider research around animal adoption and acquisition from various sources [38–41]. Lack of owner awareness around health was part of the major theme of owner expectation which led to both unexpected costs as well confusing messaging and information around infectious disease risk, with some owners feeling they were given inconsistent or incorrect information. This caused owner trust in veterinary surgeons to be eroded and led to a significant impact on owners’ mental health. Our findings support other UK research suggesting that knowledge and awareness of exotic diseases in imported dogs is poor [42]. In an EU survey of citizen awareness of zoonotic disease, most indicated that they received very little, if any, information on zoonoses although when asked what topics they would like to know more about, it was high on the list [37].

Similarly, if owners felt knowledgeable about the likely behavioural issues of IRDs and informed and supported to take these on, they were generally much more satisfied than if this was not the case. Rescue organisations were perceived to have a moral responsibility to provide accurate information and adequate post-adoption support. When some overseas rescue organisations do not have the capacity to help owners with behavioural problems or to kennel dogs, it was felt to fall on UK rescues to provide support, negatively impacting their resources.

The impact of pre-and post-adoption knowledge and support requires further investigation in order to better understand these concerns and their implications for dog welfare and owner wellbeing. Behavioural screening and assessment and the provision of accurate and detailed information about the rescue dog’s behaviour is likely fundamental to improvements in order to identify the most suitable dogs for the UK pet market and allow prospective adopters to make informed decisions. However, a predicted challenge to this could be if the dog’s behaviour changes upon import. Similarly, our findings suggest that clear, accurate guidance pre-adoption around infectious disease risks by origin, testing, results and treatment options, would be a helpful intervention and aid vets, owners and rescues in making shared decisions.

### Enforcement and regulation

The current study determined that IRDs were sometimes non-compliant with regulations and were therefore imported illegally. Dogs were being transported as fit for travel whilst heavily pregnant or as underage puppies, with incorrect or falsified paperwork, and without the correct rabies vaccination or worming status. The current commercial and non-commercial movement regulations for companion animals were thought to be ineffective and difficult to enforce and to monitor. Loopholes such as moving commercial animals (including rescue dogs) via the non-commercial pet travel rules, or by owner declarations, needs to be addressed. A lack of Border Control Posts at the most common ports of UK entry and the movement monitoring system were believed to increase the risk of non-compliance, prevent traceability and mask the dog’s rabies risk, especially if the importers do not fulfil the extra requirements necessary to bring dogs from non EU-countries. The system was perceived to suffer from lack of adequate resources to identify intelligence and perform checks, and is limited because it relies on the behaviours of people in other countries who are not within the jurisdiction of the UK to enforce compliance.

As there is no specific legislation governing UK rescues, and overseas rescues are perhaps even more diverse, it may be difficult for potential owners to assess what to look for in a “good” rescue organisation, in order to avoid incorrect practices. Limiting rehoming of imported puppies by registered and licensed charities only, with mandatory post-adoption back-up as a condition of licensing, has been suggested [43]. Similarly, the ‘Approved Importers Scheme’ was recently implemented but was not deemed a success by some stakeholders at least at the time of the interviews. Since the UK Government has left the single market, it can raise the minimum age to import puppies to six months; this method has been used successfully in other countries (e.g., USA) [44] and is widely supported as a proposal [45,46]. However, it also has the potential to restrict importation of new genetic lines of pedigree dogs, potentially increasing levels of inbreeding in UK pools. It would also only impact puppies, not adult rescue dogs as evidenced here as also experiencing significant problems.

### Limitations and future directions

This study used caregiver and other stakeholder reports and perceptions and was limited to those who volunteered their time for interview or posted on the internet forums used. Owners who post questions regarding behavioural issues in their pets are obviously having problems, but responses to their posts were wide ranging from those with both positive and negative experiences and advice to give. Those who responded to the adverts for interviews/focus groups may have been more engaged on the issues with strong views on importation; however this includes both positive and negative views on the practice, and a wide range of viewpoints were sought through our networks and reflected in our data. Due to the nature of these stakeholders, the majority of those interviewed were female. They included self-reported experiences of stakeholders such as veterinary surgeons, behaviourists and rescue workers, and the dogs presented to these groups may be more likely to be those that have more severe health behavioural, issues, requiring intervention. Actual behavioural and physiological assessments at multiple timepoints (such as in the shelter at the country of origin, during transportation, on arrival to the UK, and after various time periods situated in their new home) are still required. Whilst our findings may not be completely transferable to all views and experiences, the aim of this study was to investigate a range of the lived experience of stakeholders involved in order to illustrate their views of the welfare implications of importing rescue dogs from overseas, which this study achieved.

Whilst conducting this study we also found that policy and practice in this area changes quickly, thus some details may be applicable only to the time of the study. For example, it would be useful to conduct an assessment of the ‘Approved Importers Scheme’ to determine its effectiveness in reducing non-compliance, given that in its early stages we found perceptions that it had not been as effective as hoped, but this may have since changed. However, whilst many of the political forces governing this trade are fluid and subject to change, the underlying issues described here were believed by participants to be long-standing and consistent.

## Conclusions

The current study identified key challenges faced by dogs and their owners and other key stakeholders when importing dogs from overseas, including the short- and long-term welfare impacts on animals, and the impact on diverse dog and veterinary professionals. Negative effects of transportation and rehoming were identified, including animal behaviour and health problems and decreased mental wellbeing of staff and new owners interacting with these animals. Importantly, however, the impact of importation on individual welfare may vary. Some owners emphasized few or no issues with the importation process, health or behavioural issues in their new pets. Improved regulation is considered in dire need if the welfare of imported dogs is to be addressed and suffering minimized. Based on the experiences of stakeholders, the need to promote sector co-operation and change is recommended, as well as increased education of the general public about risks associated with imported dogs and what is good practice. There is also need for improved regulation of commercially imported dogs and wider dog-related activities/professions. In particular our evidence supports a recommendation of clearer communication avenues both within and between stakeholders, including with imported rescue dog owners. This will contribute to addressing pet owner expectations and misconceptions, and provide a starting point for improving the welfare of dogs imported into the UK and elsewhere.

## Supporting information

S1 File Interview and Focus group schedule. (DOCX)
