# Supplementary material for: “They’re just shifting a problem”: A qualitative study of stakeholder perceptions of the welfare implications of UK rescue dog importation
Source: PLoS One. 2026 Sep 9;21(9):e0343416. doi: 10.1371/journal.pone.0343416 (PMC13557394; doi:10.1371/journal.pone.0343416)
Supplement: Supplementary file 1 [file pone.0343416.s001.docx]

Supporting information:

“They’re just shifting a problem”: A qualitative study of stakeholder perceptions of the welfare implications of UK rescue dog importation

**Interview and Focus group schedule**

Version number & date: V1 10/08/22

Research ethics approval number: VREC1233a

Title of the research project: **Exploring the welfare implications of the international rehoming of dogs**

Name of researcher(s): Dr Lisa Wallis, Dr Carri Westgarth, Dr Gina Pinchbeck

The questions below are a rough guide and may be altered depending on new information arising during the project, or depending on the experiences of the participant that are being shared.

**Focus Groups – For those working/volunteering in overseas rescue dog organisations or transporters of dogs**

Each workshop (whether in person or online) will have two hosts, one to be the actual chair and the other to ask extra questions when needed, take notes and take over in case of internet issues.

- Check all consent
- Check all are aware that participation in these groups are to remain anonymous and information is not to be shared further
- Can you give examples of what you feel is good and bad practice in terms of importation of dogs for rehoming?
- What is your experience of health in imported dogs, including during transport?
- What is your experience of behaviour in imported dogs, including any impact of transport?
- Do you have any human health and safety concerns in terms of working in the area of imported dogs?
- What measures are used to protect or improve the welfare of imported dogs?
- What are the main barriers to ensuring good welfare for imported dogs?
- In your experience what was the impact of the recent ban on importation of some dogs from overseas?
- What should be done to improve welfare of both imported dogs and UK dogs?

**Interview schedule – Owners**

- Check consent
- Briefly tell me about your dog.
- How/when was your dog imported?
- Can you tell me how the dog travelled to the UK?
- Have you had a rescue dog before (from in or outside the UK?)
- Why did you choose to own this imported rescue dog?
- What have been your experiences of the health of your dog?
- What have been your experiences of the behaviour of your dog?
- If one of your friends or family was considering adopting an overseas dog, on the basis of your experiences, what advice would you give?

**Interview schedule – other stakeholders (eg vets, border control, police, UK rescue organisations)**

- Check consent
- In what capacity or circumstances do you encounter imported dogs?
- What is your experience of health in imported dogs, including during transport?
- What is your experience of behaviour of imported dogs, including any impact of transport?
- Can you give examples of what you feel is good and bad practice in terms of importation of dogs for rehoming?
- Do you have any human health and safety concerns in terms of working in the area of imported dogs?
- What measures are used to protect or improve the welfare of imported dogs?
- What are the main barriers to ensuring good welfare for imported dogs?
- What should be done to improve welfare of both imported dogs and UK dogs?
