# Supplementary material for: “They’re just shifting a problem”: A qualitative study of stakeholder perceptions of the welfare implications of UK rescue dog importation
Source: PLoS One. 2026 Sep 9;21(9):e0343416. doi: 10.1371/journal.pone.0343416 (PMC13557394; doi:10.1371/journal.pone.0343416)
Supplement: Supplementary file 2 [file pone.0343416.s002.docx]

**Major Comments**

**1. Researcher reflexivity and positionality need expansion**

The manuscript appropriately notes the multidisciplinary backgrounds of the research team; however, it would benefit from a more explicit reflexivity statement. Given the policy-relevant and potentially sensitive nature of dog importation, greater transparency regarding how researchers’ professional experiences (e.g., veterinary practice, rescue involvement, DEFRA-funded work) may have influenced data collection or interpretation would strengthen the rigor of the study. A brief paragraph outlining positionality, prior assumptions, and steps taken to minimize interpretive bias would enhance trustworthiness.

**2. Clarification on funder involvement and research independence**

The manuscript states that the study was funded by DEFRA and conducted independently, which is important to communicate. To fully reassure readers, it would be helpful to expand on:

- whether the funder had any role in shaping research questions, recruitment, or analysis
- who holds data ownership
- whether the authors retained full autonomy in interpretation and publication decisions

Providing these details will increase transparency and address potential concerns about influence on framing or findings.

**3. Sampling strategy and self-selection bias require deeper discussion**

Recruitment relied on voluntary participation via professional networks and social media. This approach is understandable for a qualitative study, but it may increase the likelihood of engaging stakeholders with strong views on importation. A more developed discussion of potential sampling biases, whether any efforts were made to capture contrasting or underrepresented viewpoints, and how such limitations affect transferability would strengthen methodological clarity.

**4. Provide additional descriptive participant information (within anonymity constraints)**

Participant characteristics are currently described primarily in terms of gender distribution. Additional aggregated information—such as years of experience, region of work, or extent of involvement with imported dogs—would help readers contextualize the dataset and evaluate transferability. Even high-level summaries would be useful and should not compromise anonymity.

**5. Improve analytic transparency regarding coding procedures**

The description of the thematic analysis is currently brief. To align with qualitative reporting standards, please expand on:

- whether a codebook was developed and iteratively refined
- the specific steps of the analytic process
- whether any inter-coder checking or collaborative coding took place
- how the research team ensured consistency and credibility (e.g., triangulation meetings, audit trails)

If formal double-coding was not undertaken, briefly explain the rationale and describe any alternative credibility strategies used.

**6. Provide clearer justification for combining interviews, focus groups, and forum posts**

The use of multiple data sources can enrich qualitative insights, but it would help readers if the manuscript were elaborated on:

- why forum data were included
- how these data complement interview and focus group material
- whether different analytic procedures were applied to each source

Clarifying this will enhance methodological coherence.

**7. Consider adding descriptive visuals to enhance readability**

While the study is qualitative, the clarity of presentation could be improved with a small number of descriptive, non-quantitative visuals, such as:

- a bar chart summarizing participant numbers by stakeholder category
- a flow diagram outlining the study design and data sources
- a thematic map illustrating relationships among main themes

These would assist readers in navigating the study without altering the qualitative nature of the research.

**Minor Comments**

**8. Ensure high-resolution versions of Figures 1 and 2**

Figures appear low-resolution in the submission files. Please ensure publication-quality versions are provided.

**9. Copyediting for clarity and flow**

A light copyedit is recommended to address occasional typographical issues and improve sentence flow.

**Overall Assessment**

This manuscript addresses a timely and important topic with clear implications for animal welfare, public health, and policy. The use of multiple qualitative data sources provides a rich foundation for exploring stakeholder perspectives, and the themes identified are relevant and thoughtfully discussed.

To maximize transparency and methodological rigor, the manuscript would benefit from additional detail regarding reflexivity, participant characteristics, sampling limitations, and analytic procedures. These enhancements would strengthen confidence in the findings and increase the manuscript’s contribution to the field.

I recommend **major revisions** at this stage.
