# Supplementary material for: “They’re just shifting a problem”: A qualitative study of stakeholder perceptions of the welfare implications of UK rescue dog importation
Source: PLoS One. 2026 Sep 9;21(9):e0343416. doi: 10.1371/journal.pone.0343416 (PMC13557394; doi:10.1371/journal.pone.0343416)
Supplement: Supplementary file 3 [file pone.0343416.s003.docx]

**RESPONSE TO REVIEWERS AND EDITORS**

**PONE-D-25-53004**

**“They’re just shifting a problem”: A qualitative study of stakeholder perceptions of the welfare implications of UK rescue dog importation**

PLOS One
When submitting your revision, we need you to address these additional requirements.

1.Please ensure that your manuscript meets PLOS ONE's style requirements, including those for file naming. The PLOS ONE style templates can be found at

<https://journals.plos.org/plosone/s/file?id=wjVg/PLOSOne_formatting_sample_main_body.pdf> and

[https://journals.plos.org/plosone/s/file?id=ba62/PLOSOne_formatting_sample_title_authors_affiliations.pdf](https://track.editorialmanager.com/CL0/https:%2F%2Fjournals.plos.org%2Fplosone%2Fs%2Ffile%3Fid=ba62%2FPLOSOne_formatting_sample_title_authors_affiliations.pdf/1/010f019b258bd7c8-57c8a3af-87e0-4234-8c64-8855a66e2803-000000/s459eGPrXOPPmT0nnkP2br4hmK13__4q73nmYUaApgw=241)

RESPONSE: As far as we are aware it meets these guidelines, apart from we have now centered the title, and corrected the double spacing on the references.

2. Thank you for stating the following financial disclosure:

“The research presented in this paper was funded by the Department of the Environment, Farming and Rural Affairs (Defra) project (34061) “EXPLORING THE WELFARE IMPLICATIONS OF THE INTERNATIONAL REHOMING OF DOGS.””

Please state what role the funders took in the study.  If the funders had no role, please state: "The funders had no role in study design, data collection and analysis, decision to publish, or preparation of the manuscript."

If this statement is not correct you must amend it as needed.

Please include this amended Role of Funder statement in your cover letter; we will change the online submission form on your behalf.

3. Thank you for stating the following in the Competing Interests section:

“This research was funded by DEFRA including the post of LW. CW has conducted consulting for MARS Petcare, Royal Canin and Forthglade. LB runs Brucella canis RVN, a social media-based page to raise awareness of Brucella canis (BC) among stakeholders, improve access to evidence-based information, support owners of BC positive dogs, and runs workshops for rescue organisations to improve pre-importation protocols for BC. JS works as a Clinical Research Manager at VetPartners Limited. The remaining authors declare that the research was conducted in the absence of any commercial or financial relationships that could be construed as a potential conflict of interest. The funders did not play any role in the study design, collection, analysis, and interpretation of data, or in the decision to submit to PLOS One.”

We note that one or more of the authors are employed by a commercial company: VetPartners Limited

1. Please provide an amended Funding Statement declaring this commercial affiliation, as well as a statement regarding the Role of Funders in your study. If the funding organization did not play a role in the study design, data collection and analysis, decision to publish, or preparation of the manuscript and only provided financial support in the form of authors' salaries and/or research materials, please review your statements relating to the author contributions, and ensure you have specifically and accurately indicated the role(s) that these authors had in your study. You can update author roles in the Author Contributions section of the online submission form.

Please also include the following statement within your amended Funding Statement.

“The funder provided support in the form of salaries for authors [insert relevant initials], but did not have any additional role in the study design, data collection and analysis, decision to publish, or preparation of the manuscript. The specific roles of these authors are articulated in the ‘author contributions’ section.”

If your commercial affiliation did play a role in your study, please state and explain this role within your updated Funding Statement.

2. Please also provide an updated Competing Interests Statement declaring this commercial affiliation along with any other relevant declarations relating to employment, consultancy, patents, products in development, or marketed products, etc.

Within your Competing Interests Statement, please confirm that this commercial affiliation does not alter your adherence to all PLOS ONE policies on sharing data and materials by including the following statement: "This does not alter our adherence to  PLOS ONE policies on sharing data and materials.” (as detailed online in our guide for authors <http://journals.plos.org/plosone/s/competing-interests>) . If this adherence statement is not accurate and  there are restrictions on sharing of data and/or materials, please state these. Please note that we cannot proceed with consideration of your article until this information has been declared.

Please include both an updated Funding Statement and Competing Interests Statement in your cover letter. We will change the online submission form on your behalf.

RESPONSE

Updated funding statement:

The research presented in this paper was funded by the Department of the Environment, Farming and Rural Affairs (Defra) project (34061) “EXPLORING THE WELFARE IMPLICATIONS OF THE INTERNATIONAL REHOMING OF DOGS”, namely the salary of LW. The funders had no role in study design, data collection and analysis, or decision to publish, and provided limited comment to the drafted manuscript. VetPartners provided support in the form of salary for author JS, but did not have any additional role in the study design, data collection and analysis, decision to publish, or preparation of the manuscript. The specific roles of these authors are articulated in the ‘author contributions’ section.

Updated competing interests statement:

This research was funded by DEFRA including the post of LW. The funders had no role in study design, data collection and analysis, or decision to publish, and provided limited comment to the drafted manuscript. CW has conducted consulting for MARS Petcare, Royal Canin and Forthglade. LB runs Brucella canis RVN, a social media-based page to raise awareness of Brucella canis (BC) among stakeholders, improve access to evidence-based information, support owners of BC positive dogs, and runs workshops for rescue organisations to improve pre-importation protocols for BC. JS works as a Clinical Research Manager at VetPartners Limited. This does not alter our adherence to PLOS ONE policies on sharing data and materials. The remaining authors declare that the research was conducted in the absence of any commercial or financial relationships that could be construed as a potential conflict of interest.”

4. We note that you have indicated that there are restrictions to data sharing for this study. PLOS only allows data to be available upon request if there are legal or ethical restrictions on sharing data publicly. For more information on unacceptable data access restrictions, please see <http://journals.plos.org/plosone/s/data-availability#loc-unacceptable-data-access-restrictions>.

Before we proceed with your manuscript, please address the following prompts:

a) If there are ethical or legal restrictions on sharing a de-identified data set, please explain them in detail (e.g., data contain potentially identifying or sensitive patient information, data are owned by a third-party organization, etc.) and who has imposed them (e.g., a Research Ethics Committee or Institutional Review Board, etc.). Please also provide contact information for a data access committee, ethics committee, or other institutional body to which data requests may be sent.

b) If there are no restrictions, please upload the minimal anonymized data set necessary to replicate your study findings to a stable, public repository and provide us with the relevant URLs, DOIs, or accession numbers. For a list of recommended repositories, please see

<https://journals.plos.org/plosone/s/recommended-repositories>. You also have the option of uploading the data as Supporting Information files, but we would recommend depositing data directly to a data repository if possible.

We will update your Data Availability statement on your behalf to reflect the information you provide.

RESPONSE:

We have clarified this to:

“Data cannot be shared publicly due to containing potentially identifying and sensitive information, given the controversial nature of the topic and unique roles of many stakeholders. Ethical approval (by the University of Liverpool Infection, Veterinary and Ecological Sciences Ethics Committee) was only given for participants to consent for their anonymised data to be archived for future-related research projects under restricted access on application to the project authors. Thus, anonymised transcripts are available for researchers for bone fide research project purposes by application to the University of Liverpool Infection, Veterinary and Ecological Sciences Ethics Committee ([ivesethics@liverpool.ac.uk](mailto:ivesethics@liverpool.ac.uk)) and subject to approval by them, and the equivalent Institutional Review Board of the researchers requesting the data.”

5. If the reviewer comments include a recommendation to cite specific previously published works, please review and evaluate these publications to determine whether they are relevant and should be cited. There is no requirement to cite these works unless the editor has indicated otherwise.

Additional Editor Comments:
Pay particular attention to comments from reviewer no. 2. He has raised pertinent issues that need to be addressed before the article can be considered for publication.

Reviewers' comments:

Reviewer's Responses to Questions

**Comments to the Author**

1. Is the manuscript technically sound, and do the data support the conclusions?

The manuscript must describe a technically sound piece of scientific research with data that supports the conclusions. Experiments must have been conducted rigorously, with appropriate controls, replication, and sample sizes. The conclusions must be drawn appropriately based on the data presented.

Reviewer #1: Yes

Reviewer #2: Partly

2. Has the statistical analysis been performed appropriately and rigorously?

Reviewer #1: Yes

Reviewer #2: N/A

3. Have the authors made all data underlying the findings in their manuscript fully available?

The [PLOS Data policy](http://track.editorialmanager.com/CL0/http:%2F%2Fwww.plosone.org%2Fstatic%2Fpolicies.action%23sharing/1/010f019b258bd7c8-57c8a3af-87e0-4234-8c64-8855a66e2803-000000/UUa-gmlc3EGSPraYPhiNHk1x5DFtrcxZletCf6OUWnk=241) requires authors to make all data underlying the findings described in their manuscript fully available without restriction, with rare exception (please refer to the Data Availability Statement in the manuscript PDF file). The data should be provided as part of the manuscript or its supporting information, or deposited to a public repository. For example, in addition to summary statistics, the data points behind means, medians and variance measures should be available. If there are restrictions on publicly sharing data—e.g. participant privacy or use of data from a third party—those must be specified.

Reviewer #1: Yes

Reviewer #2: No

4. Is the manuscript presented in an intelligible fashion and written in standard English?

PLOS ONE does not copyedit accepted manuscripts, so the language in submitted articles must be clear, correct, and unambiguous. Any typographical or grammatical errors should be corrected at revision, so please note any specific errors here.

Reviewer #1: Yes

Reviewer #2: Yes

5. Review Comments to the Author

Please use the space provided to explain your answers to the questions above. You may also include additional comments for the author, including concerns about dual publication, research ethics, or publication ethics. (Please upload your review as an attachment if it exceeds 20,000 characters)

Reviewer #1: As an adoptor of a Romanian rescue dog with behavioural issues, I'm pleased to see this research, which strikes me as unusually objective: negative opinions about foreign rescues are generally strongly expressed among the veterinary community and often lack understanding of the nuances of each situation or focus on infectious disease without considering the real welfare issues (travel, the dog and humans' mental adjustment etc). Unbiased, thorough research like this, which investigates the lived experiences of a range of people involved in the lives of these rescue dogs, is therefore much needed and I hope this paper will be impactful. The authors, especially Carri Westgarth and Lisa Wallis, have published extensively on related topics as well as teaching One Health and the Human-Animal Bond, and I was especially pleased to see VN Louise Buckley involved. Louise has a unique understanding of the bonds between owners and their foreign rescues based on her work on the BC Facebook Group, and is vocal about her resistance to the “fear-based” treatment of dogs suspected of having Brucella canis. I'm very happy to see this 'scaremongering' addressed in the paper.

RESPONSE: We thank you for your thoughtful and enthusiastic review in particular concerning the objectivity and nuance of the research.

The methodology appears sound: 30 interviews, 4 focus groups and 18 pubic forums provide extensive data and the qualitative approach seems appropriate to conduct a more nuanced investigation. The thematic analysis is a useful way to organise the data and I'm pleased to see two themes on transport, a huge welfare concern often neglected in discussions around this topic, which has tended to focus on infectious disease (the 'fit to travel' theme contained some particularly shocking revelations). Interesting that 83% of participants were female but perhaps not suprising - recent research has shown that the primary caregiver for a family pet tends to be the woman in the home (see Belshaw 'More than just one man and his dog: The many impacts of puppy acquisition on the mental health of families including children in the UK' - maybe this could be cited here?)

RESPONSE: It is already well known that animal caregivers, animal care professionals such as veterinary, rescue and training, are more likely to be female, as well as respondents to survey/interview requests in general, and specifically in the area of human-animal interaction. Further, because this study involved interviews with a range of different types of stakeholders rather than just one such as dog owners, we chose not to add the multiple citations required to support all these sources of likely female bias. Further, due to the nature of this particular study also involving other professions such as animal transporters, government worker and trading standards officer, we actually had a relatively high number of male participants at 17%. We have however added a sentence in the discussion to highlight the female majority “Due to the nature of these stakeholders, the majority of those interviewed were female.”

In the Introduction, the authors list possible reasons for the increasing popularity of importing rescue dogs. I wonder if there is any evidence that relatively strict restrictions on local UK adoptions (i.e., not being able to take a Dogs Trust or RSPCA dog because a garden is too small, there are children in the house etc) is driving people to adopt from abroad? Anectodally, this is why I adopted overseas and why most others I know with foreign rescues did so.

RESPONSE: Yes, these were evidenced in our previous survey of adopters (Norman et al., 2020) but were less reported reasons than the ones we state in the introduction when we summarise that paper (“social media advertising, wanting to rescue rather than fuel breeding, and the belief that foreign dogs suffer more than dogs in UK rescue centres”)

The welfare problems revealed by this research are extensive and important, but I would like to see a little more on the positive experiences - there are a few lines and one quote (517-524) but I'm certain that more participants would have expressed positivity about rescues who are now living happy, healthy lives in the UK (and the conclusion seems to suggest this). I know that this is not the overall objective of the paper but a balanced approach which acknowledges that - if rescue organisations provided more accurate information, better matching processes and greater adopter support - foreign rescue dogs CAN experience positive welfare here, would be beneficial.

RESPONSE:

Due to the nature of the research, to investigate the welfare challenges, most of the discussion in the interviews concerned this. We do present positive experiences under the theme of owner expectations with a supporting quote, and in response to your comment have added two more quotes to strengthen this aspect of the findings.

Nevertheless, where owners and caregivers were willing and able to commit to the IRD, they found the experience rewarding, despite the difficulties encountered. Hard work, patience and long-term commitment were recognised as necessary by owners to progress and reach a suitable standard with their IRD, and for some the journey was ongoing.

*‘When he first arrived, he was worried about everything, but with time and patience he has expanded his comfort zone, and it has been wonderful to watch him grow.’ (Forum participant)*

*"Most rewarding thing I have ever done. I love my two to bits. I have had them for 6 years now and every time I look at their happy faces I am filled with gratitude. It's been far from easy, but I wouldn't change them for anything." (Forum participant)*

*"If you give them the right home, where they have got time, patience, love and kindness. They will eventually turn around. […] As long as they have a relationship with someone, a bond with someone, they turn out to be really nice pets for those people and those homes." (Dog behaviourist interview participant, charity volunteer and owner).*

Really good to see the issues around compliance, regulation and bisecurity addressed in this paper, topics which need to be brought to public attention.

Overall, I congratulate the authors on this insightful paper which reveals the many flaws in the current overseas adoption system. I hope it will help tighten up regulations so that overseas rescue dogs do not end up experiencing worse welfare than they did in their home countries.

RESPONSE:

Many thanks for your thoughtful and positive comments.

Reviewer #2: Overall Assessment
This manuscript addresses a timely and important topic with clear implications for animal welfare, public health, and policy. The use of multiple qualitative data sources provides a rich foundation for exploring stakeholder perspectives, and the themes identified are relevant and thoughtfully discussed.
To maximize transparency and methodological rigor, the manuscript would benefit from additional detail regarding reflexivity, participant characteristics, sampling limitations, and analytic procedures. These enhancements would strengthen confidence in the findings and increase the manuscript’s contribution to the field.
I recommend major revisions at this stage.

**Major Comments**

**1. Researcher reflexivity and positionality need expansion**

The manuscript appropriately notes the multidisciplinary backgrounds of the research team; however, it would benefit from a more explicit reflexivity statement. Given the policy-relevant and potentially sensitive nature of dog importation, greater transparency regarding how researchers’ professional experiences (e.g., veterinary practice, rescue involvement, DEFRA-funded work) may have influenced data collection or interpretation would strengthen the rigor of the study. A brief paragraph outlining positionality, prior assumptions, and steps taken to minimize interpretive bias would enhance trustworthiness.

RESPONSE:

Some more information was previously provided in the competing interests statement and methods, but we have expanded and moved this specifically into a positionality section.

We did already say in the data analysis section “Initial themes were generated and similarities and differences identified by LW and AM (interviews/focus groups) and AM (forum analysis) in regular conversation with CW. These were then further refined in collaboration with the other authors”

We have clarified this to

“The study was conducted by a multidisciplinary academic team, with a wide range of backgrounds, experiences and views regarding imported rescue dogs, including owning them and providing veterinary care to them. Online interviews were conducted by LW who is a female qualitative and quantitative postdoctoral researcher, non-vet with a background in animal behaviour, who has imported her owned dogs into the UK when moving job locations. Focus groups were conducted by LW and CW. CW is the Principal Investigator of the study, a non-vet female qualitative and quantitative researcher with a background in animal behaviour and human public health, who owns multiple rescue dogs including a rescue breeding dog thought to be from Ireland/Northern Ireland, and has also worked for a UK animal rescue charity. The selection and analysis of forum data was conducted by AM a veterinary undergraduate student interested in the topic, who also analysed the trainer/behaviourist focus group. The project was supported by collaborators GP, a female veterinary epidemiologist, JS a veterinary researcher who has worked in shelter medicine, and LB a veterinary nurse academic who owns multiple imported rescue dogs and runs a *Brucella canis* advisory service. The immediate data collection and analysis team (CW, LW and AM) entered the study with mixed views regarding importation of rescue dogs, all recognising both positive and negative aspects to the practice. The wider supporting team all had veterinary backgrounds and a diverse range of views and practices, generally either supporting or opposing the importation of rescue dogs. In summary, we expected to find a number of human health and animal welfare challenges regarding imported rescue dogs, in particular concerning behaviour and infectious disease, and a number of practices occurring around importation that impacted these outcomes both positively and negatively.”

**2. Clarification on funder involvement and research independence**

The manuscript states that the study was funded by DEFRA and conducted independently, which is important to communicate. To fully reassure readers, it would be helpful to expand on:

- whether the funder had any role in shaping research questions, recruitment, or analysis
- who holds data ownership
- whether the authors retained full autonomy in interpretation and publication decisions

Providing these details will increase transparency and address potential concerns about influence on framing or findings.

RESPONSE: These are all in the funding and competing interests sections (see above) that we presume that this reviewer did not have access to. We hope that seeing these now satisfied this.

**3. Sampling strategy and self-selection bias require deeper discussion**

Recruitment relied on voluntary participation via professional networks and social media. This approach is understandable for a qualitative study, but it may increase the likelihood of engaging stakeholders with strong views on importation. A more developed discussion of potential sampling biases, whether any efforts were made to capture contrasting or underrepresented viewpoints, and how such limitations affect transferability would strengthen methodological clarity.

RESPONSE: We have expanded this part of the limitations discussion, which now reads:

“This study used caregiver and other stakeholder reports and perceptions and was limited to those who volunteered their time for interview or posted on the internet forums used. Owners who post questions regarding behavioural issues in their pets are obviously having problems, but responses to their posts were wide ranging from those with both positive and negative experiences and advice to give. Those who responded to the adverts for interviews/focus groups may have been more engaged on the issues with strong views on importation; however this includes both positive and negative views on the practice, and a wide range of viewpoints were sought through our networks and reflected in our data. Due to the nature of these stakeholders, the majority of those interviewed were female. They included self-reported experiences of stakeholders such as veterinary surgeons, behaviourists and rescue workers, and the dogs presented to these groups may be more likely to be those that have more severe health behavioural, issues, requiring intervention. Actual behavioural and physiological assessments at multiple timepoints (such as in the shelter at the country of origin, during transportation, on arrival to the UK, and after various time periods situated in their new home) are still required. Whilst our findings may not be completely transferable to all views and experiences, the aim of this study was to investigate a range of the lived experience of stakeholders involved in order to illustrate their views of the welfare implications of importing rescue dogs from overseas, which this study achieved.”

**4. Provide additional descriptive participant information (within anonymity constraints)**

Participant characteristics are currently described primarily in terms of gender distribution. Additional aggregated information—such as years of experience, region of work, or extent of involvement with imported dogs—would help readers contextualize the dataset and evaluate transferability. Even high-level summaries would be useful and should not compromise anonymity.

RESPONSE: We did not collect years of experience as demographic data. The regions of work and type of involvement in imported dogs are already presented in Table 1. We have added an assessment on overall views on IRD gathered from recruitment data:

“From initial recruitment data collected, the participants selected appeared to have positive views (15%), neutral/mixed views (11%), negative views (22%) or not provided (52%).”

The only other data we collected was about the dogs they owned, which we don’t feel is relevant to report here.

**5. Improve analytic transparency regarding coding procedures**

The description of the thematic analysis is currently brief. To align with qualitative reporting standards, please expand on:

- whether a codebook was developed and iteratively refined
- the specific steps of the analytic process
- whether any inter-coder checking or collaborative coding took place
- how the research team ensured consistency and credibility (e.g., triangulation meetings, audit trails)

If formal double-coding was not undertaken, briefly explain the rationale and describe any alternative credibility strategies used.

RESPONSE: Double coding was not used due to time constraints but two people coded different parts of the data in regular three-way discussion with the primary supervisor.

The analysis section has been updated and expanded.

“Audio recordings of the interviews/focus groups were transcribed either professionally or within Microsoft Teams. Thematic analysis of the interview/focus group transcripts (LW and AM) and discussion threads (AM) was conducted employing inductive coding managed in NVIVO software. Concurrent interviews and analysis were conducted until it was felt that a reasonable level of data saturation was reached to be able to present a number of clear themes and sub-themes [18, 19]. Braun and Clarke’s (2006) six-phase process was used: 1) familiarising with the data; 2) generating initial codes; 3) searching for themes; 4) reviewing themes; 5) defining and naming themes; 6) producing the report [20]. Initial codes and themes were generated and similarities and differences identified by AM (forum analysis of behavioural concerns) supervised by LW and CW, who wrote her own report on these for her undergraduate studies. Separately and concurrently, LW analysed interviews/focus groups, supervised by CW, with further assistance from AM for analysing the behaviourist/trainer focus group. In order to challenge assumptions and consider alternative explanations, increasing rigor and further minimising the effect of the positionality of the researcher [21], the development of codes and themes were discussed in weekly conversations with CW; LW also attended the supervisory meetings of AM. Themes were then further refined in collaboration with the other authors during wider team discussions every 2-3 months and during drafting of the project report for the funder, which formed the basis of this paper. This enabled the creation of main themes, which encompassed the key subjects of the discussions, and sub-themes describing more detail within these. Quotes have been anonymised; given verbatim for those interviewed but slightly modified for ethical reasons for those taken from public forums in order to preserve anonymity. Due to time constraints, participants were not invited to provide feedback on transcripts or findings.”

**6. Provide clearer justification for combining interviews, focus groups, and forum posts**

The use of multiple data sources can enrich qualitative insights, but it would help readers if the manuscript were elaborated on:

- why forum data were included
- how these data complement interview and focus group material
- whether different analytic procedures were applied to each source

Clarifying this will enhance methodological coherence.

RESPONSE:

We have added “Forum data were included as an additional accessible resource in order to encompass a larger sample of views around IRD and their behaviour than possible through interviews and focus groups alone. As the data is naturally occurring and not influenced by the presence of researchers it therefore may be less affected by social desirability bias which can affect surveys and interviews [18].”

We have also since clarified in the methods how analytic procedures applied to each source were the same but conducted in the first instance by a different researcher, but with regular team discussions.

**7. Consider adding descriptive visuals to enhance readability**

While the study is qualitative, the clarity of presentation could be improved with a small number of descriptive, non-quantitative visuals, such as:

- a bar chart summarizing participant numbers by stakeholder category
- a flow diagram outlining the study design and data sources
- a thematic map illustrating relationships among main themes

These would assist readers in navigating the study without altering the qualitative nature of the research.

RESPONSE:

We feel that “a bar chart summarizing participant numbers by stakeholder category” would duplicate Table 1, although we have added percentage to this table for clarity.

We attempted to create a flow diagram (new Fig 1) but this is challenging to map out in much more detail than already given in the text/table 1 because of the multiple identities/stakeholder categories many people fell under (for example founding a rescue, being a trainer, and owning imported rescue dogs).

The overall thematic map is contained in what was previously Figure 1 (now Figure 2) already.

For brevity we left out further thematic maps other than what was previously Figure 2 but have now added three further schematic theme map figures (3-5).

Fig 3

Fig 4

Fig 5

Fig 6, was previously Fig 2
