# Supplementary material for: “They’re just shifting a problem”: A qualitative study of stakeholder perceptions of the welfare implications of UK rescue dog importation
Source: PLoS One. 2026 Sep 9;21(9):e0343416. doi: 10.1371/journal.pone.0343416 (PMC13557394; doi:10.1371/journal.pone.0343416)
Supplement: Peer review history

# Peer review: Decision Letter 0

**DOI:** 10.1371/journal.pone.0343416.r001

**Authors:** Eric Ogola

16 Dec 2025

Dear Dr.  Westgarth,

Thank you for submitting your manuscript to PLOS ONE. After careful consideration, we feel that it has merit but does not fully meet PLOS ONE’s publication criteria as it currently stands. Therefore, we invite you to submit a revised version of the manuscript that addresses the points raised during the review process.

Please submit your revised manuscript by Jan 29 2026 11:59PM. If you will need more time than this to complete your revisions, please reply to this message or contact the journal office at plosone@plos.org. When you're ready to submit your revision, log on to https://www.editorialmanager.com/pone/ and select the 'Submissions Needing Revision' folder to locate your manuscript file.

- A letter that responds to each point raised by the academic editor and reviewer(s). You should upload this letter as a separate file labeled 'Response to Reviewers'.
- A marked-up copy of your manuscript that highlights changes made to the original version. You should upload this as a separate file labeled 'Revised Manuscript with Track Changes'.
- An unmarked version of your revised paper without tracked changes. You should upload this as a separate file labeled 'Manuscript'.

If you would like to make changes to your financial disclosure, please include your updated statement in your cover letter. Guidelines for resubmitting your figure files are available below the reviewer comments at the end of this letter.

If applicable, we recommend that you deposit your laboratory protocols in protocols.io to enhance the reproducibility of your results. Protocols.io assigns your protocol its own identifier (DOI) so that it can be cited independently in the future. For instructions see: https://journals.plos.org/plosone/s/submission-guidelines#loc-laboratory-protocols . Additionally, PLOS ONE offers an option for publishing peer-reviewed Lab Protocol articles, which describe protocols hosted on protocols.io. Read more information on sharing protocols at https://plos.org/protocols?utm_medium=editorial-email&utm_source=authorletters&utm_campaign=protocols .

We look forward to receiving your revised manuscript.

Kind regards,

Eric Ogola, MPH

Academic Editor

PLOS One

Journal Requirements:

When submitting your revision, we need you to address these additional requirements.

1.Please ensure that your manuscript meets PLOS ONE's style requirements, including those for file naming. The PLOS ONE style templates can be found at

https://journals.plos.org/plosone/s/file?id=wjVg/PLOSOne_formatting_sample_main_body.pdf and

https://journals.plos.org/plosone/s/file?id=ba62/PLOSOne_formatting_sample_title_authors_affiliations.pdf

2. Thank you for stating the following financial disclosure:

“The research presented in this paper was funded by the Department of the Environment, Farming and Rural Affairs (Defra) project (34061) “EXPLORING THE WELFARE IMPLICATIONS OF THE INTERNATIONAL REHOMING OF DOGS.””

Please state what role the funders took in the study.  If the funders had no role, please state: "The funders had no role in study design, data collection and analysis, decision to publish, or preparation of the manuscript."

If this statement is not correct you must amend it as needed.

Please include this amended Role of Funder statement in your cover letter; we will change the online submission form on your behalf.

3. Thank you for stating the following in the Competing Interests section:

“This research was funded by DEFRA including the post of LW. CW has conducted consulting for MARS Petcare, Royal Canin and Forthglade. LB runs Brucella canis RVN, a social media-based page to raise awareness of Brucella canis (BC) among stakeholders, improve access to evidence-based information, support owners of BC positive dogs, and runs workshops for rescue organisations to improve pre-importation protocols for BC. JS works as a Clinical Research Manager at VetPartners Limited. The remaining authors declare that the research was conducted in the absence of any commercial or financial relationships that could be construed as a potential conflict of interest. The funders did not play any role in the study design, collection, analysis, and interpretation of data, or in the decision to submit to PLOS One.”

We note that one or more of the authors are employed by a commercial company: VetPartners Limited

1. Please provide an amended Funding Statement declaring this commercial affiliation, as well as a statement regarding the Role of Funders in your study. If the funding organization did not play a role in the study design, data collection and analysis, decision to publish, or preparation of the manuscript and only provided financial support in the form of authors' salaries and/or research materials, please review your statements relating to the author contributions, and ensure you have specifically and accurately indicated the role(s) that these authors had in your study. You can update author roles in the Author Contributions section of the online submission form.

Please also include the following statement within your amended Funding Statement.

“The funder provided support in the form of salaries for authors [insert relevant initials], but did not have any additional role in the study design, data collection and analysis, decision to publish, or preparation of the manuscript. The specific roles of these authors are articulated in the ‘author contributions’ section.”

If your commercial affiliation did play a role in your study, please state and explain this role within your updated Funding Statement.

2. Please also provide an updated Competing Interests Statement declaring this commercial affiliation along with any other relevant declarations relating to employment, consultancy, patents, products in development, or marketed products, etc.

Within your Competing Interests Statement, please confirm that this commercial affiliation does not alter your adherence to all PLOS ONE policies on sharing data and materials by including the following statement: "This does not alter our adherence to  PLOS ONE policies on sharing data and materials.” (as detailed online in our guide for authors http://journals.plos.org/plosone/s/competing-interests) . If this adherence statement is not accurate and  there are restrictions on sharing of data and/or materials, please state these. Please note that we cannot proceed with consideration of your article until this information has been declared.

Please include both an updated Funding Statement and Competing Interests Statement in your cover letter. We will change the online submission form on your behalf.

4. We note that you have indicated that there are restrictions to data sharing for this study. PLOS only allows data to be available upon request if there are legal or ethical restrictions on sharing data publicly. For more information on unacceptable data access restrictions, please see http://journals.plos.org/plosone/s/data-availability#loc-unacceptable-data-access-restrictions.

Before we proceed with your manuscript, please address the following prompts:

a) If there are ethical or legal restrictions on sharing a de-identified data set, please explain them in detail (e.g., data contain potentially identifying or sensitive patient information, data are owned by a third-party organization, etc.) and who has imposed them (e.g., a Research Ethics Committee or Institutional Review Board, etc.). Please also provide contact information for a data access committee, ethics committee, or other institutional body to which data requests may be sent.

b) If there are no restrictions, please upload the minimal anonymized data set necessary to replicate your study findings to a stable, public repository and provide us with the relevant URLs, DOIs, or accession numbers. For a list of recommended repositories, please see

https://journals.plos.org/plosone/s/recommended-repositories. You also have the option of uploading the data as Supporting Information files, but we would recommend depositing data directly to a data repository if possible.

We will update your Data Availability statement on your behalf to reflect the information you provide.

5. If the reviewer comments include a recommendation to cite specific previously published works, please review and evaluate these publications to determine whether they are relevant and should be cited. There is no requirement to cite these works unless the editor has indicated otherwise.

Additional Editor Comments:

Pay particular attention to comments from reviewer no. 2. He has raised pertinent issues that need to be addressed before the article can be considered for publication.

[Note: HTML markup is below. Please do not edit.]

Reviewers' comments:

Reviewer's Responses to Questions

**Comments to the Author**

1. Is the manuscript technically sound, and do the data support the conclusions?

Reviewer #1: Yes

Reviewer #2: Partly

**********

2. Has the statistical analysis been performed appropriately and rigorously?

Reviewer #1: Yes

Reviewer #2: N/A

**********

3. Have the authors made all data underlying the findings in their manuscript fully available?

The PLOS Data policy

Reviewer #1: Yes

Reviewer #2: No

**********

4. Is the manuscript presented in an intelligible fashion and written in standard English?

Reviewer #1: Yes

Reviewer #2: Yes

**********

Reviewer #1: As an adoptor of a Romanian rescue dog with behavioural issues, I'm pleased to see this research, which strikes me as unusually objective: negative opinions about foreign rescues are generally strongly expressed among the veterinary community and often lack understanding of the nuances of each situation or focus on infectious disease without considering the real welfare issues (travel, the dog and humans' mental adjustment etc). Unbiased, thorough research like this, which investigates the lived experiences of a range of people involved in the lives of these rescue dogs, is therefore much needed and I hope this paper will be impactful. The authors, especially Carri Westgarth and Lisa Wallis, have published extensively on related topics as well as teaching One Health and the Human-Animal Bond, and I was especially pleased to see VN Louise Buckley involved. Louise has a unique understanding of the bonds between owners and their foreign rescues based on her work on the BC Facebook Group, and is vocal about her resistance to the “fear-based” treatment of dogs suspected of having Brucella canis. I'm very happy to see this 'scaremongering' addressed in the paper.

The methodology appears sound: 30 interviews, 4 focus groups and 18 pubic forums provide extensive data and the qualitative approach seems appropriate to conduct a more nuanced investigation. The thematic analysis is a useful way to organise the data and I'm pleased to see two themes on transport, a huge welfare concern often neglected in discussions around this topic, which has tended to focus on infectious disease (the 'fit to travel' theme contained some particularly shocking revelations). Interesting that 83% of participants were female but perhaps not suprising - recent research has shown that the primary caregiver for a family pet tends to be the woman in the home (see Belshaw 'More than just one man and his dog: The many impacts of puppy acquisition on the mental health of families including children in the UK' - maybe this could be cited here?)

In the Introduction, the authors list possible reasons for the increasing popularity of importing rescue dogs. I wonder if there is any evidence that relatively strict restrictions on local UK adoptions (i.e., not being able to take a Dogs Trust or RSPCA dog because a garden is too small, there are children in the house etc) is driving people to adopt from abroad? Anectodally, this is why I adopted overseas and why most others I know with foreign rescues did so.

The welfare problems revealed by this research are extensive and important, but I would like to see a little more on the positive experiences - there are a few lines and one quote (517-524) but I'm certain that more partipants would have expressed positivity about rescues who are now living happy, healthy lives in the UK (and the conclusion seems to suggest this). I know that this is not the overall objective of the paper but a balanced approach which acknowledges that - if rescue organisations provided more accurate information, better matching processes and greater adopter support - foreign rescue dogs CAN experience positive welfare here, would be beneficial.

Really good to see the issues around compliance, regulation and bisecurity addressed in this paper, topics which need to be brought to public attention.

Overall, I congratulate the authors on this insightful paper which reveals the many flaws in the current overseas adoption system. I hope it will help tighten up regulations so that overseas rescue dogs do not end up experiencing worse welfare than they did in their home countries.

Reviewer #2: Overall Assessment

This manuscript addresses a timely and important topic with clear implications for animal welfare, public health, and policy. The use of multiple qualitative data sources provides a rich foundation for exploring stakeholder perspectives, and the themes identified are relevant and thoughtfully discussed.

To maximize transparency and methodological rigor, the manuscript would benefit from additional detail regarding reflexivity, participant characteristics, sampling limitations, and analytic procedures. These enhancements would strengthen confidence in the findings and increase the manuscript’s contribution to the field.

I recommend major revisions at this stage.

**********

what does this mean? ). If published, this will include your full peer review and any attached files.

If you choose “no”, your identity will remain anonymous but your review may still be made public.

**Do you want your identity to be public for this peer review?**  For information about this choice, including consent withdrawal, please see our Privacy Policy

Reviewer #1: **Yes:**   Alice Oven

Reviewer #2: No

**********

[NOTE: If reviewer comments were submitted as an attachment file, they will be attached to this email and accessible via the submission site. Please log into your account, locate the manuscript record, and check for the action link "View Attachments". If this link does not appear, there are no attachment files.]

To ensure your figures meet our technical requirements, please review our figure guidelines: https://journals.plos.org/plosone/s/figures

You may also use PLOS’s free figure tool, NAAS, to help you prepare publication quality figures: https://journals.plos.org/plosone/s/figures#loc-tools-for-figure-preparation.

NAAS will assess whether your figures meet our technical requirements by comparing each figure against our figure specifications.

Attachment Submitted filename: PONE-D-25-53004_review_2.docx

# Author comment: Author response to Decision Letter 1

**DOI:** 10.1371/journal.pone.0343416.r002

28 Jan 2026

RESPONSE TO REVIEWERS AND EDITORS

PONE-D-25-53004

“They’re just shifting a problem”: A qualitative study of stakeholder perceptions of the welfare implications of UK rescue dog importation

PLOS One

When submitting your revision, we need you to address these additional requirements.

1.Please ensure that your manuscript meets PLOS ONE's style requirements, including those for file naming. The PLOS ONE style templates can be found at

https://journals.plos.org/plosone/s/file?id=wjVg/PLOSOne_formatting_sample_main_body.pdf and

https://journals.plos.org/plosone/s/file?id=ba62/PLOSOne_formatting_sample_title_authors_affiliations.pdf

RESPONSE: As far as we are aware it meets these guidelines, apart from we have now centered the title, and corrected the double spacing on the references.

2. Thank you for stating the following financial disclosure:

“The research presented in this paper was funded by the Department of the Environment, Farming and Rural Affairs (Defra) project (34061) “EXPLORING THE WELFARE IMPLICATIONS OF THE INTERNATIONAL REHOMING OF DOGS.””

Please state what role the funders took in the study. If the funders had no role, please state: "The funders had no role in study design, data collection and analysis, decision to publish, or preparation of the manuscript."

If this statement is not correct you must amend it as needed.

Please include this amended Role of Funder statement in your cover letter; we will change the online submission form on your behalf.

3. Thank you for stating the following in the Competing Interests section:

“This research was funded by DEFRA including the post of LW. CW has conducted consulting for MARS Petcare, Royal Canin and Forthglade. LB runs Brucella canis RVN, a social media-based page to raise awareness of Brucella canis (BC) among stakeholders, improve access to evidence-based information, support owners of BC positive dogs, and runs workshops for rescue organisations to improve pre-importation protocols for BC. JS works as a Clinical Research Manager at VetPartners Limited. The remaining authors declare that the research was conducted in the absence of any commercial or financial relationships that could be construed as a potential conflict of interest. The funders did not play any role in the study design, collection, analysis, and interpretation of data, or in the decision to submit to PLOS One.”

We note that one or more of the authors are employed by a commercial company: VetPartners Limited

1. Please provide an amended Funding Statement declaring this commercial affiliation, as well as a statement regarding the Role of Funders in your study. If the funding organization did not play a role in the study design, data collection and analysis, decision to publish, or preparation of the manuscript and only provided financial support in the form of authors' salaries and/or research materials, please review your statements relating to the author contributions, and ensure you have specifically and accurately indicated the role(s) that these authors had in your study. You can update author roles in the Author Contributions section of the online submission form.

Please also include the following statement within your amended Funding Statement.

“The funder provided support in the form of salaries for authors [insert relevant initials], but did not have any additional role in the study design, data collection and analysis, decision to publish, or preparation of the manuscript. The specific roles of these authors are articulated in the ‘author contributions’ section.”

If your commercial affiliation did play a role in your study, please state and explain this role within your updated Funding Statement.

2. Please also provide an updated Competing Interests Statement declaring this commercial affiliation along with any other relevant declarations relating to employment, consultancy, patents, products in development, or marketed products, etc.

Within your Competing Interests Statement, please confirm that this commercial affiliation does not alter your adherence to all PLOS ONE policies on sharing data and materials by including the following statement: "This does not alter our adherence to PLOS ONE policies on sharing data and materials.” (as detailed online in our guide for authors http://journals.plos.org/plosone/s/competing-interests) . If this adherence statement is not accurate and there are restrictions on sharing of data and/or materials, please state these. Please note that we cannot proceed with consideration of your article until this information has been declared.

Please include both an updated Funding Statement and Competing Interests Statement in your cover letter. We will change the online submission form on your behalf.

RESPONSE

Updated funding statement:

The research presented in this paper was funded by the Department of the Environment, Farming and Rural Affairs (Defra) project (34061) “EXPLORING THE WELFARE IMPLICATIONS OF THE INTERNATIONAL REHOMING OF DOGS”, namely the salary of LW. The funders had no role in study design, data collection and analysis, or decision to publish, and provided limited comment to the drafted manuscript. VetPartners provided support in the form of salary for author JS, but did not have any additional role in the study design, data collection and analysis, decision to publish, or preparation of the manuscript. The specific roles of these authors are articulated in the ‘author contributions’ section.

Updated competing interests statement:

This research was funded by DEFRA including the post of LW. The funders had no role in study design, data collection and analysis, or decision to publish, and provided limited comment to the drafted manuscript. CW has conducted consulting for MARS Petcare, Royal Canin and Forthglade. LB runs Brucella canis RVN, a social media-based page to raise awareness of Brucella canis (BC) among stakeholders, improve access to evidence-based information, support owners of BC positive dogs, and runs workshops for rescue organisations to improve pre-importation protocols for BC. JS works as a Clinical Research Manager at VetPartners Limited. This does not alter our adherence to PLOS ONE policies on sharing data and materials. The remaining authors declare that the research was conducted in the absence of any commercial or financial relationships that could be construed as a potential conflict of interest.”

4. We note that you have indicated that there are restrictions to data sharing for this study. PLOS only allows data to be available upon request if there are legal or ethical restrictions on sharing data publicly. For more information on unacceptable data access restrictions, please see http://journals.plos.org/plosone/s/data-availability#loc-unacceptable-data-access-restrictions.

Before we proceed with your manuscript, please address the following prompts:

a) If there are ethical or legal restrictions on sharing a de-identified data set, please explain them in detail (e.g., data contain potentially identifying or sensitive patient information, data are owned by a third-party organization, etc.) and who has imposed them (e.g., a Research Ethics Committee or Institutional Review Board, etc.). Please also provide contact information for a data access committee, ethics committee, or other institutional body to which data requests may be sent.

b) If there are no restrictions, please upload the minimal anonymized data set necessary to replicate your study findings to a stable, public repository and provide us with the relevant URLs, DOIs, or accession numbers. For a list of recommended repositories, please see

https://journals.plos.org/plosone/s/recommended-repositories. You also have the option of uploading the data as Supporting Information files, but we would recommend depositing data directly to a data repository if possible.

We will update your Data Availability statement on your behalf to reflect the information you provide.

RESPONSE:

We have clarified this to:

“Data cannot be shared publicly due to containing potentially identifying and sensitive information, given the controversial nature of the topic and unique roles of many stakeholders. Ethical approval (by the University of Liverpool Infection, Veterinary and Ecological Sciences Ethics Committee) was only given for participants to consent for their anonymised data to be archived for future-related research projects under restricted access on application to the project authors. Thus, anonymised transcripts are available for researchers for bone fide research project purposes by application to the University of Liverpool Infection, Veterinary and Ecological Sciences Ethics Committee (ivesethics@liverpool.ac.uk) and subject to approval by them, and the equivalent Institutional Review Board of the researchers requesting the data.”

5. If the reviewer comments include a recommendation to cite specific previously published works, please review and evaluate these publications to determine whether they are relevant and should be cited. There is no requirement to cite these works unless the editor has indicated otherwise.

Additional Editor Comments:

Pay particular attention to comments from reviewer no. 2. He has raised pertinent issues that need to be addressed before the article can be considered for publication.

Reviewers' comments:

Reviewer's Responses to Questions

Comments to the Author

1. Is the manuscript technically sound, and do the data support the conclusions?

The manuscript must describe a technically sound piece of scientific research with data that supports the conclusions. Experiments must have been conducted rigorously, with appropriate controls, replication, and sample sizes. The conclusions must be drawn appropriately based on the data presented.

Reviewer #1: Yes

Reviewer #2: Partly

________________________________________

2. Has the statistical analysis been performed appropriately and rigorously?

Reviewer #1: Yes

Reviewer #2: N/A

________________________________________

3. Have the authors made all data underlying the findings in their manuscript fully available?

The PLOS Data policy requires authors to make all data underlying the findings described in their manuscript fully available without restriction, with rare exception (please refer to the Data Availability Statement in the manuscript PDF file). The data should be provided as part of the manuscript or its supporting information, or deposited to a public repository. For example, in addition to summary statistics, the data points behind means, medians and variance measures should be available. If there are restrictions on publicly sharing data—e.g. participant privacy or use of data from a third party—those must be specified.

Reviewer #1: Yes

Reviewer #2: No

________________________________________

4. Is the manuscript presented in an intelligible fashion and written in standard English?

PLOS ONE does not copyedit accepted manuscripts, so the language in submitted articles must be clear, correct, and unambiguous. Any typographical or grammatical errors should be corrected at revision, so please note any specific errors here.

Reviewer #1: Yes

Reviewer #2: Yes

________________________________________

5. Review Comments to the Author

Please use the space provided to explain your answers to the questions above. You may also include additional comments for the author, including concerns about dual publication, research ethics, or publication ethics. (Please upload your review as an attachment if it exceeds 20,000 characters)

Reviewer #1: As an adoptor of a Romanian rescue dog with behavioural issues, I'm pleased to see this research, which strikes me as unusually objective: negative opinions about foreign rescues are generally strongly expressed among the veterinary community and often lack understanding of the nuances of each situation or focus on infectious disease without considering the real welfare issues (travel, the dog and humans' mental adjustment etc). Unbiased, thorough research like this, which investigates the lived experiences of a range of people involved in the lives of these rescue dogs, is therefore much needed and I hope this paper will be impactful. The authors, especially Carri Westgarth and Lisa Wallis, have published extensively on related topics as well as teaching One Health and the Human-Animal Bond, and I was especially pleased to see VN Louise Buckley involved. Louise has a unique understanding of the bonds between owners and their foreign rescues based on her work on the BC Facebook Group, and is vocal about her resistance to the “fear-based” treatment of dogs suspected of having Brucella canis. I'm very happy to see this 'scaremongering' addressed in the paper.

RESPONSE: We thank you for your thoughtful and enthusiastic review in particular concerning the objectivity and nuance of the research.

The methodology appears sound: 30 interviews, 4 focus groups and 18 pubic forums provide extensive data and the qualitative approach seems appropriate to conduct a more nuanced investigation. The thematic analysis is a useful way to organise the data and I'm pleased to see two themes on transport, a huge welfare concern often neglected in discussions around this topic, which has tended to focus on infectious disease (the 'fit to travel' theme contained some particularly shocking revelations). Interesting that 83% of participants were female but perhaps not suprising - recent research has shown that the primary caregiver for a family pet tends to be the woman in the home (see Belshaw 'More than just one man and his dog: The many impacts of puppy acquisition on the mental health of families including children in the UK' - maybe this could be cited here?)

RESPONSE: It is already well known that animal caregivers, animal care professionals such as veterinary, rescue and training, are more likely to be female, as well as respondents to survey/interview requests in general, and specifically in the area of human-animal interaction. Further, because this study involved interviews with a range of different types of stakeholders rather than just one such as dog owners, we chose not to add the multiple citations required to support all these sources of likely female bias. Further, due to the nature of this particular study also involving other professions such as animal transporters, government worker and trading standards officer, we actually had a relatively high number of male participants at 17%. We have however added a sentence in the discussion to highlight the female majority “Due to the nature of these stakeholders, the majority of those interviewed were female.”

In the Introduction, the authors list possible reasons for the increasing popularity of importing rescue dogs. I wonder if there is any evidence that relatively strict restrictions on local UK adoptions (i.e., not being able to take a Dogs Trust or RSPCA dog because a garden is too small, there are children in the house etc) is driving people to adopt from abroad? Anectodally, this is why I adopted overseas and why most others I know with foreign rescues did so.

RESPONSE: Yes, these were evidenced in our previous survey of adopters (Norman et al., 2020) but were less reported reasons than the ones we state in the introduction when we summarise that paper (“social media advertising, wanting to rescue rather than fuel breeding, and the belief that foreign dogs suffer more than dogs in UK rescue centres”)

The welfare problems revealed by this research are extensive and important, but I would like to see a little more on the positive experiences - there are a few lines and one quote (517-524) but I'm certain that more participants would have expressed positivity about rescues who are now living happy, healthy lives in the UK (and the conclusion seems to suggest this). I know that this is not the overall objective of the paper but a balanced approach which acknowledges that - if rescue organisations provided more accurate information, better matching processes and greater adopter support - foreign rescue dogs CAN experience positive welfare here, would be beneficial.

RESPONSE:

Due to the nature of the research, to investigate the welfare challenges, most of the discussion in

Attachment Submitted filename: Reviewer response 1 IRD PLOSOne.docx

# Editor report: Decision Letter 1

**DOI:** 10.1371/journal.pone.0343416.r003

**Authors:** Eric Ogola

5 Feb 2026

“They’re just shifting a problem”: A qualitative study of stakeholder perceptions of the welfare implications of UK rescue dog importation

PONE-D-25-53004R1

Dear Dr. Westgarth,

We’re pleased to inform you that your manuscript has been judged scientifically suitable for publication and will be formally accepted for publication once it meets all outstanding technical requirements.

Within one week, you’ll receive an e-mail detailing the required amendments. When these have been addressed, you’ll receive a formal acceptance letter and your manuscript will be scheduled for publication.

An invoice will be generated when your article is formally accepted. Please note, if your institution has a publishing partnership with PLOS and your article meets the relevant criteria, all or part of your publication costs will be covered. Please make sure your user information is up-to-date by logging into Editorial Manager at Editorial Manager®  and clicking the ‘Update My Information' link at the top of the page. For questions related to billing, please contact billing support .

If your institution or institutions have a press office, please notify them about your upcoming paper to help maximize its impact. If they’ll be preparing press materials, please inform our press team as soon as possible -- no later than 48 hours after receiving the formal acceptance. Your manuscript will remain under strict press embargo until 2 pm Eastern Time on the date of publication. For more information, please contact onepress@plos.org.

Kind regards,

Eric Ogola, MPH

Academic Editor

PLOS One

Additional Editor Comments (optional):

Reviewers' comments:

# Editor report: Acceptance letter

**DOI:** 10.1371/journal.pone.0343416.r004

**Authors:** Eric Ogola

PONE-D-25-53004R1

PLOS One

Dear Dr. Westgarth,

I'm pleased to inform you that your manuscript has been deemed suitable for publication in PLOS One. Congratulations! Your manuscript is now being handed over to our production team.

At this stage, our production department will prepare your paper for publication. This includes ensuring the following:

* All references, tables, and figures are properly cited

* All relevant supporting information is included in the manuscript submission,

* There are no issues that prevent the paper from being properly typeset

You will receive further instructions from the production team, including instructions on how to review your proof when it is ready. Please keep in mind that we are working through a large volume of accepted articles, so please give us a few days to review your paper and let you know the next and final steps.

Lastly, if your institution or institutions have a press office, please let them know about your upcoming paper now to help maximize its impact. If they'll be preparing press materials, please inform our press team within the next 48 hours. Your manuscript will remain under strict press embargo until 2 pm Eastern Time on the date of publication. For more information, please contact onepress@plos.org.

You will receive an invoice from PLOS for your publication fee after your manuscript has reached the completed accept phase. If you receive an email requesting payment before acceptance or for any other service, this may be a phishing scheme. Learn how to identify phishing emails and protect your accounts at https://explore.plos.org/phishing.

If we can help with anything else, please email us at customercare@plos.org.

Thank you for submitting your work to PLOS ONE and supporting open access.

Kind regards,

PLOS ONE Editorial Office Staff

on behalf of

Dr. Eric Ogola

Academic Editor

PLOS One
